# 24 million years of pollination interaction between European linden flowers and bumble bees

**DOI:** 10.1111/nph.70531

**Published:** 2025-09-22

**Authors:** Christian Geier, Michael S. Engel, Johannes M. Bouchal, Silvia Ulrich, Jürg Schönenberger, Dieter Uhl, Torsten Wappler, Sonja Wedmann, Loup Boudet, Friðgeir Grímsson

**Affiliations:** ^1^ Department of Botany and Biodiversity Research University of Vienna Rennweg 14 1030 Vienna Austria; ^2^ Division of Invertebrate Zoology American Museum of Natural History 200 Central Park West New York NY 10024‐5192 USA; ^3^ Department of Historical Archaeology Austrian Academy of Sciences (OeAW), Austrian Archaeological Institute (OeAI) Dominikanerbastei 16 1010 Vienna Austria; ^4^ Senckenberg Forschungsinstitut und Naturmuseum Frankfurt Senckenberganlage 25 60325 Frankfurt am Main Germany; ^5^ Department of Natural History Hessisches Landesmuseum Darmstadt Friedensplatz 1 64283 Darmstadt Germany; ^6^ Rheinische Friedrich‐Wilhelms Universität Bonn Bonner Institut für Organismische Biologie: Abteilung V Paläontologie Nußallee 8 53115 Bonn Germany; ^7^ Senckenberg Forschungsinstitut und Naturmuseum Frankfurt Senckenberg Forschungsstation Grube Messel Markstraße 35 64409 Messel Germany

**Keywords:** *Bombus*, Cenozoic, fossil *in situ* pollen, Hymenoptera, Malvaceae, plant‐insect interactions, *Tilia*, Tilioideae

## Abstract

- Pollination is the most common insect–plant mutualism, binding them in a co‐evolutionary framework. Historic evidence of this interaction can be partly inferred from time‐calibrated molecular phylogenies of plant and insect lineages or directly from fossils. Fossils providing such evidence are sparse and only a few fossiliferous localities offer adequate preservation of both flowers and insects.
- We screened fossil flowers and bees from the Late Oligocene (Chattian) of Enspel, Germany, using white and fluorescent light, followed by palynological sampling and detailed investigation. Flowers are identified via pollen and floral morphology in comparison with modern taxa. The bumble bees are described and placed into a morphological framework with extant congeners. The pollination biology of extant *Tilia* is summarized and complemented by field observations.
- We report the new fossil species *Tilia magnasepala* C. Geier et Schönenb. sp. nov. (Tilioideae, Malvaceae), *Bombus* (*Kronobombus*) *messegus* Engel et Wappler, sp. nov., and *Bombus* (*Timebombus*) *palaeocrater* Engel et Wappler, sp. nov. (Apidae: Bombini).
- The presence of the same *Tilia* pollen *in situ* in flowers and adhering to the exterior of the bumble bees provides direct evidence for their interaction and the role of *Bombus* as a pollinator for *Tilia* by at least the Late Oligocene and persisting to the present.

## Introduction

The angiosperm subfamily Tilioideae Arnott is monophyletic, nested within Malvaceae *s.l*., and comprises the genera *Tilia* L., *Craigia* W.W.Sm. & W.E.Evans, and *Mortoniodendron* Standl. & Steyerm (Nyffeler *et al*., 2005; Le Péchon & Gigord, 2014; Colli‐Silva *et al*., 2025). Tilioideae are estimated to have diverged from the remainder of the family during the Late Cretaceous, *c*. 79.2 million years ago (Ma) (85.6–72.6 Ma; Santonian or Campanian), and further diversified c. 72.7 Ma (80.4–65.4 Ma; Campanian or Maastrichtian; see table S4 in Hernández‐Gutiérrez & Magallón, 2019), suggesting that its modern genera trace back to the onset of the Cenozoic or that at least the stem lineages to these genera were present during the Late Cretaceous and earliest Paleogene. Currently, *Tilia* comprises *c*. 30 species divided into four sections, *Tilia* sect. Anastraea Engl., *T*. sect. Astrophilyra Engl., *T*. sect. Endochrysea H.T. Chang, and *T*. sect. Henryana Pigott (Pigott, 2012; POWO, 2024; but see fig. 1 in Xie *et al*. (2023) where they recognize two sections with one divided into three subsections resulting in four clades). The genus has a predominantly temperate to subtropical Northern Hemispheric distribution, extending into tropical regimes, with species in both North and Central America (2–4 spp.), Europe and West Asia (5–6 spp.), and East Asia (14–17 spp.; Pigott, 2012). *Tilia* has an extensive Cenozoic fossil record composed mostly of leaves and pollen (*e.g*. Muller, 1981; Krutzsch, 2004; Stuchlik *et al*., 2014; Hinsley, 2020; and references therein). Fossil pollen grains represent the earliest records, suggesting the presence of crown‐group *Tilia* by the Paleocene (*e.g*. Mai, 1961; Muller, 1981; Krutzsch, 2004; and references therein). The earliest fruiting structures in the fossil record are bracts from the late Eocene of North America (Manchester, 1994), but comparable fossils occur only later in Europe, specifically in the early Oligocene (Hably *et al*., 2000; Kvaček & Walther, 2004). Until now, only a single flower and a flower bud from the late Eocene/early Oligocene of the United States (Hall & Swain, 1971; Manchester, 1994), and bundles of stamens from the Oligocene of England (Chandler, 1957) have been described. Otherwise, the fossil record is devoid of reproductive structures. The flower–insect interactions between several present‐day species of *Tilia* have been investigated (Anderson, 1976; Corbet *et al*., 1979; Chung & Kim, 1984; Pawlikowski, 2010; Pigott, 2012; Koch & Stevenson, 2017; Jacquemart *et al*., 2018) indicating that even though flowers are visited by various insects, the main pollinators today are bees.

Here, we report a unique discovery of several fossil flowers and buds of *Tilia* with *in situ* pollen as well as several fossil bees, representing two species of bumble bees (genus *Bombus* Latreille), with adhering pollen from *Tilia*, all preserved as compressions from the late Oligocene (Chattian) of Enspel, Germany. The fossils are described and compared with flowers/buds of extant *Tilia* as well as other fossil flowers/buds of Malvaceae currently known from the geological record. The *in situ* pollen grains from the fossil flowers/buds of *Tilia* and those adhering to the fossil bees are described in detail using light microscopy (LM) and scanning electron microscopy (SEM) and transmission electron microscopy (TEM). The pollen from the fossil flowers/buds and the bees is compared with both extant and fossil pollen of *Tilia* as well as to similar pollen types from other relevant extant/fossil/extinct taxa. Our findings are also discussed in relation to the currently accepted global fossil record of *Tilia* and *Bombus*, the previously documented macro‐ and dispersed palynoflora from the Enspel site, and current knowledge on extant flower‐insect interactions between *Tilia* and their pollinators.

## Materials and Methods

### Fossil material

**Fig. 1:**
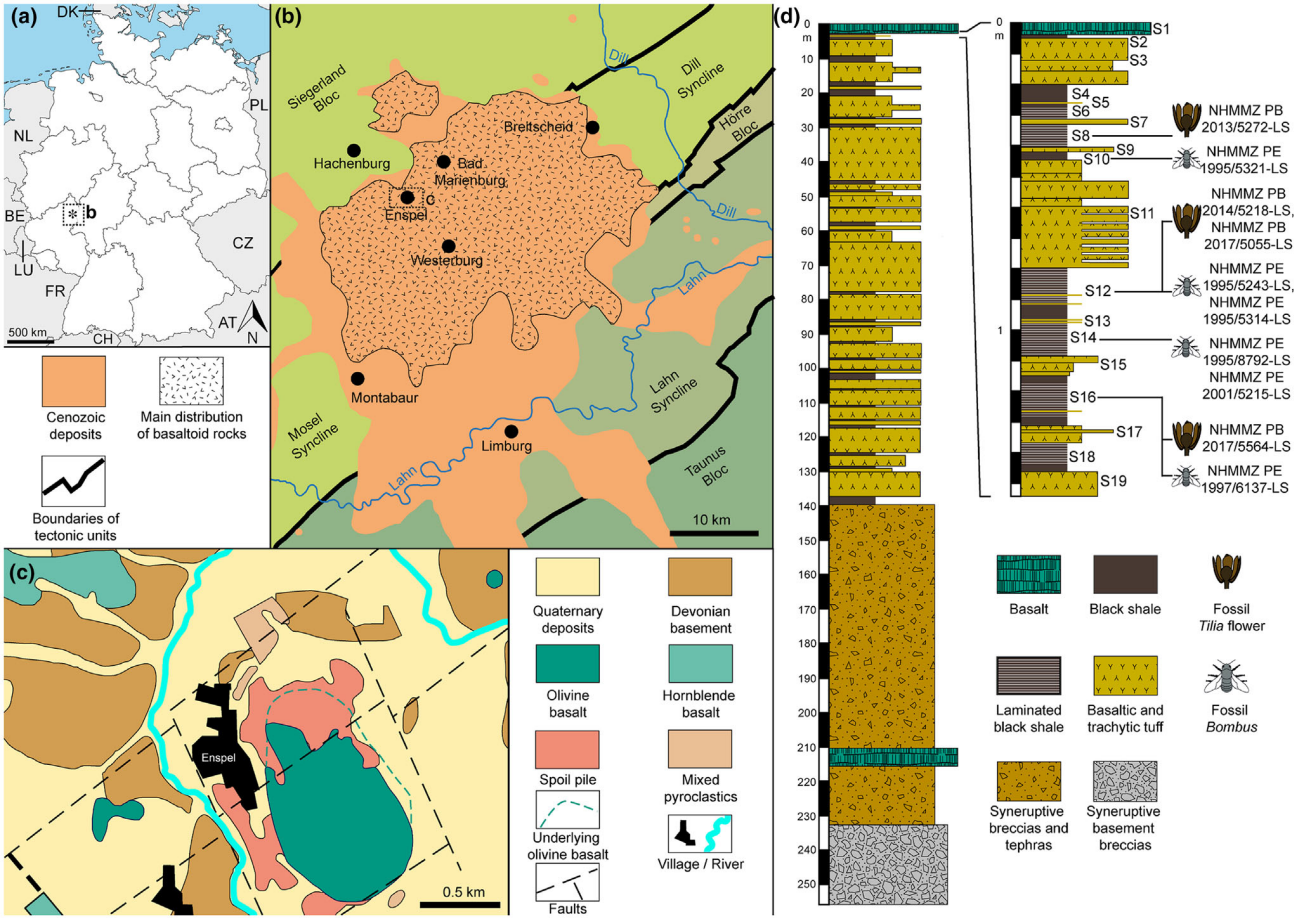
Geographical and geological maps of the study site. (**a**) Location of Enspel in Rhineland‐Palatinate, western Germany. (**b, c**) Geological formations around Enspel. (**d**) Stratigraphic column of the Enspel sediments based on drill cores (modified from Wuttke *et al.*, 2024). AT, Austria; BE, Belgium; CH, Switzerland; CZ, Czech Republic; DK, Denmark; FR, France; LU, Luxembourg; NL, Netherlands; PL, Poland.

Material reported herein is deposited in the Naturhistorisches Museum Mainz/Landessammlung für Naturkunde Rheinland‐Pfalz (NHMMZ). We screened 127 fossil flowers, inflorescences, and flower buds, and 22 fossil Hymenoptera from the late Oligocene of Enspel for *in situ* and/or adhered pollen. Out of these, four flowers and six bumble bees (Apinae: Bombini) preserved pollen of Tilioideae. The fossil flowers and buds, NHMMZ PB 2013/5272‐LS (G29/S8), NHMMZ PB 2014/5218‐LS (G29/S12u), NHMMZ PB 2017/5055‐LS (G31/S12), NHMMZ PB 2017/5564‐LS (G30/S16u), and the fossil bees, NHMMZ PE 1995/5243‐LS (G3/S12), NHMMZ PE 1995/5314‐LS (G2/S12), NHMMZ PE 1995/5321‐LS (G2/S10), NHMMZ PE 1995/8792‐LS (G5/S14), NHMMZ PE 1997/6137‐LS (G10/S16), and NHMMZ PE 2001/5215‐LS (G19B/S14) were collected between the years 1991 and 2017 during excavations of the oil shales at Enspel (Fig. 1), Germany, by the then Landesamt für Denkmalpflege Rheinland‐Pfalz/Referat Erdgeschichte, now GDKE RLP, Direktion Landesarchäologie/Erdgeschichtliche Denkmalpflege. All of the specimens were recovered from the uppermost 1.5 m of the Enspel sedimentary section, and the respective digging site (G) and oil shale layer (S) are given in brackets following the repository number (*sensu* Felder *et al*., 1998; Schindler & Wuttke, 2015).

### Geographical and geological setting

The Enspel site is located below the former (now removed by mining activity) Stöffel hill southeast of the village of Enspel in the Westerwald Mountains, Germany (UTM 32 U E: 421561.486; N: 5607702.328). The area is part of the High Westerwald volcanic field that was active during the Paleogene (Fig. 1a–c). The sediments at Enspel, representing the Enspel Formation (Fm), were formed inside a volcanic crater, accumulating within a maar‐like crater lake. The Enspel Fm is covered by massive basalt, resulting from a basalt flow into the lake that stopped sedimentary deposition, heated, and thermally changed the top‐most layers of the sedimentary succession (Fig. 1d). Radiometric dating of volcanic rocks under‐ and overlaying the Enspel Fm suggests an age range between 24.79 ± 0.05 and 24.56 ± 0.04 Ma for the fossiliferous sediments (Mertz *et al*., 2007). As the fossil flowers/buds and bees described herein were collected in the uppermost part of the Enspel Fm, close to the overlying basalt, their age is probably *c*. 24.56 ± 0.04 Ma. For further information on the geology and paleontology of the Enspel site, refer to Wedmann (2000), Schindler & Wuttke (2010, 2015), Wedmann *et al*. (2010), Schäfer *et al*. (2011), Uhl *et al*. (2024), and references therein.

### Extraction and preparation of fossil pollen from flowers/buds/insects

Both fossil flowers/buds and bees were examined, documented, and sampled with a stereomicroscope, equipped with epifluorescence illumination. The flowers/buds/insects were photographed with both white and fluorescence light. Pollen grains were extracted *in situ* from anthers or adhered to petals and/or sepals or insects (Grímsson *et al*., 2021) and processed for combined LM and SEM analysis by bleaching (Geier *et al*., 2023) and acetolysis (Halbritter *et al*., 2018). The extracted pollen grains were then investigated with both LM and SEM using the ‘Single‐grain method’ of Zetter (1989). In addition, TEM was performed following the protocol by Ulrich & Grímsson (2020) and Grímsson *et al*. (2021).

## Results

### Systematic Paleontology

Kingdom Plantae Haeckel, 1866

Clade Spermatophyta Willkomm, 1854

Division Angiospermae Hermann, 1690 *sensu* Brown, 1827 *ex* Mabberley, 2000

Order Malvales Juss. *ex* Bercht. and J.Presl, 1820

Family Malvaceae Juss., 1789

Subfamily Tilioideae Arn., 1832

Genus *Tilia* L., 1754

**Fig. 2:**
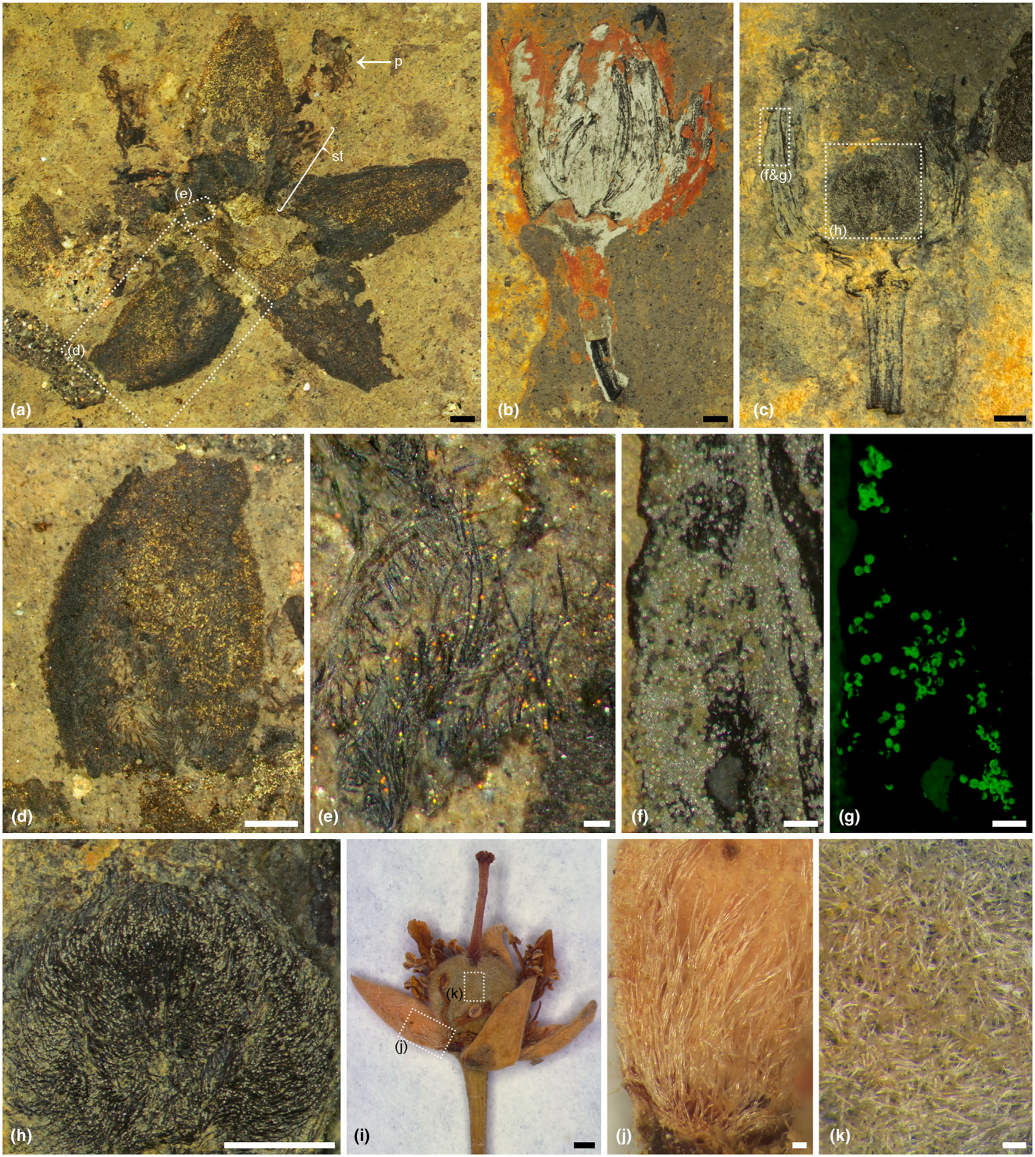
Flowers of *Tilia magnasepala* sp. nov. from the late Oligocene (Chattian) of Enspel, Germany, in different views. (**a**) NHMMZ PB 2017/5055‐LS (holotype), flower in frontal view; (**b**) NHMMZ PB 2014/5218‐LS (paratype), flower in lateral view; (**c**) NHMMZ PB 2017/5564‐LS (paratype) flower in lateral view, longitudinal section; (**d**) detail image of a sepal from the flower in (**a**); (**e**) detail of the flower in (**a**), trichomes at the base of a sepal; (**f, g**) detail of the flower in (**c**), showing area in white light (**f**) and fluorescence light (**g**), pollen fluoresces strongly; (**h**) detail of the flower in (**c**) showing the trichome‐covered superior ovary; (**i**) dry flower of extant *Tilia americana* (Cody & Spicer, Canada, WU 0151993); (**j**) detail of the flower in (**i**), long simple trichomatous nectaries at the base of the sepal; (**k**) detail of the flower in (**i**), short trichomes covering the ovary. p, petal; st, stamen. Bars, 1 mm (**a, b, c, d, h, i**), 0.1 mm (**e, f, g, j, k**).

**Fig. 3:**
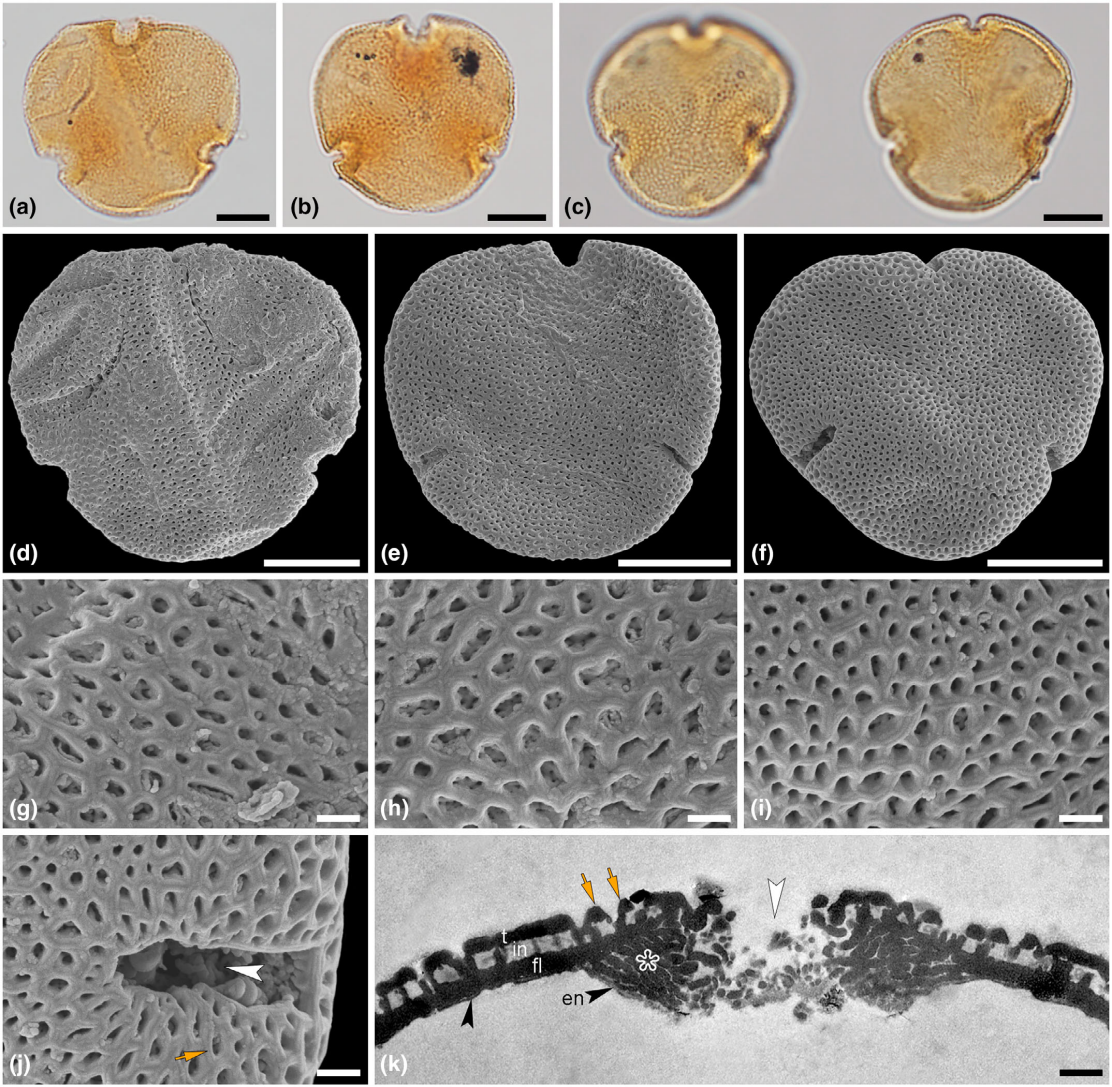
*In situ* pollen extracted from flowers of *Tilia magnasepala* sp. nov. from the Oligocene of Enspel. Light micrographs (**a–c**), scanning electron micrographs (**d–j**), and transmission electron micrograph (**k**). (**a, d, g**) NHMMZ PB 2017/5055‐LS (Fig. 2a; holotype); (**b, e, h**) NHMMZ PB 2014/5218‐LS (Fig. 2b; paratype); (**c, f, i–k**) NHMMZ PB 2017/5564‐LS (Fig. 2c; paratype). Same pollen grains in light microscopy (LM) and SEM (**a, d; b, e; c, f**); (**g–i**) close‐ups of reticulate and striate exine surface; (**j**) aperture; (**k**) ultra‐section through aperture and adjacent interapertural wall. Notes: thin, continuous compact to spongy endexine (black arrowheads, en) thicker toward aperture area (white arrowhead), ektexine with semitectate tectum (t), columellate infratectum (in), thick, continuous‐compact foot layer (fl) thicker and lamellated in aperture area (white asterisk), note faint striate supratectal ornamentation (orange arrows). Bars, 10 μm (**a–f**), 1 μm (**g–k**).

*Tilia magnasepala* C.Geier et Schönenb. sp. nov. (Figs 2, 3, Supporting Information S1).

*Holotype*: NHMMZ PB 2017/5055‐LS (Fig. 2a).

*Paratypes*: NHMMZ PB 2014/5218‐LS (Fig. 2b), NHMMZ PB 2017/5564‐LS (Fig. 2c), NHMMZ PB 2013/5272‐LS (Fig. S1a).

*Type locality*: Stöffel quarry, Enspel, Rhineland‐Palatinate, Germany.

*Type stratum*: Enspel Fm, Layer S12, digging site G31 (Schindler & Wuttke, 2015).

*Age*: 24.56 ± 0.04 Ma, Chattian, Oligocene (Mertz *et al*., 2007).

*Etymology*: The specific epithet is a combination of the Latin prefix *magna*–, meaning ‘long, large’, and the Latin plural *sēpala* for ‘sepals’. The name refers to the relatively large sepals, which are almost the same size as (and larger than) the petals. This is rare among extant *Tilia* flowers, where the petals are usually much larger than the sepals.

*Diagnosis*: Pentamerous, hermaphroditic flowers; calyx and corolla of similar dimensions, sepals ovate‐triangular, sepals with dense indumentum along margins, nectary at base of sepals formed by long, singular or fascicular trichomes; androecium formed by multiple stamens with anthers containing *Tilia*‐type pollen (*Intratriporopollenites instructus*); gynoecium superior, five‐carpellate, ovary spherical, tomentose.

*Description*: Flower pentamerous, actinomorphic, hermaphroditic, hypogynous; flower including perianth and pedicel 10.4–15.4 mm long (Fig. 2b,c), pedicel 4–6.7 mm long and 0.8–1.2 mm wide; perianth in lateral view 6.7–8.8 mm long and 6.2–8.7 mm wide (Fig. 2b,c), calyx diameter in top view 15.4–17.9 mm (Fig. 2a); sepals 6.1–6.7 mm long, 3.8–4.1 mm wide (Fig. 2a,d), ovate to triangular, internal margins of different coloration covered by trichomes, adaxial surface covered with simple trichomes, especially at base (Fig. 2d,e), basal trichomes up to 0.5 mm long and sometimes in pairs (Fig. 2e); attachment area of sepals forming a 2.4 mm wide and 0.7 mm long scar at receptacle (Fig. 2c); only remnants of petals preserved, 5.8–6.6 mm long and 1.5–2 mm wide (Fig. 2a) or compressed together with other flower organs like stamens (Fig. 2f), potentially staminodes, or sepals, hard to distinguish (Fig. 2b,c); number of stamens at least five, 2.6–3.6 mm long, anthers *c*. 1.3 mm long; gynoecium consisting of a superior, spherical ovary, 2–3 mm wide and 1.5–2.6 mm long (Fig. 2a,c), densely covered with simple trichomes (Fig. 2h), style and stigma missing, two faint longitudinal ridges visible. All flowers with *in situ* and/or adhered pollen. *In situ* pollen (Fig. 2f,g), monad, oblate, convex‐triangular to circular in polar view, elliptic in equatorial view, radially symmetrical (Figs 3, S2–S4); medium‐sized, polar axis 17–22 μm (LM), equatorial diameter 31–42 μm in LM, 27–36 μm in SEM (Fig. 3a–f); tricolporate, brevicolporate, planaperturate, lolongate ectoaperture (brevicolpus), circular endoaperture (porus), nexine thickened around apertures (costae), 2.1–3.8 μm thick and 8–9 μm wide (LM; Fig. 3a–c); exine in interapertural areas 1.3–1.8 μm thick (LM), sexine as thick or slightly thicker than nexine; sculpture reticulate (LM; Fig. 3a–c), microreticulate and perforate (SEM) sculpture uniform across the whole pollen grain, muri with a faint striate suprasculpture (Fig. 3d–j), striae running in groups of 3–5 parallel to the orientation of the muri, proximal hemisphere showing a finer reticulum than distal hemisphere (SEM). Pollen wall in TEM (Fig. 3k) has an overall thickness of 1.13–1.29 μm; columellate‐tectate ektexine 0.94–1.09 μm thick; semitectum 0.27–0.33 μm thick, columellate infratectum 0.39–0.42 μm thick, continuous‐compact foot layer 0.32–0.48 μm thick, continuous to spongy endexine *c*. 0.06 μm thin; foot layer and endexine increasing in thickness around apertures (Fig. 3k); foot layer thickening up to 1.9 μm toward apertures, gradually transitioning to a lamellate structure; endexine thickening up to 0.6 μm at aperture; striate supratectal ornamentation seen in SEM also visible in TEM in cross‐sections of pollen wall (Fig. 3k).

**Fig. 4:**
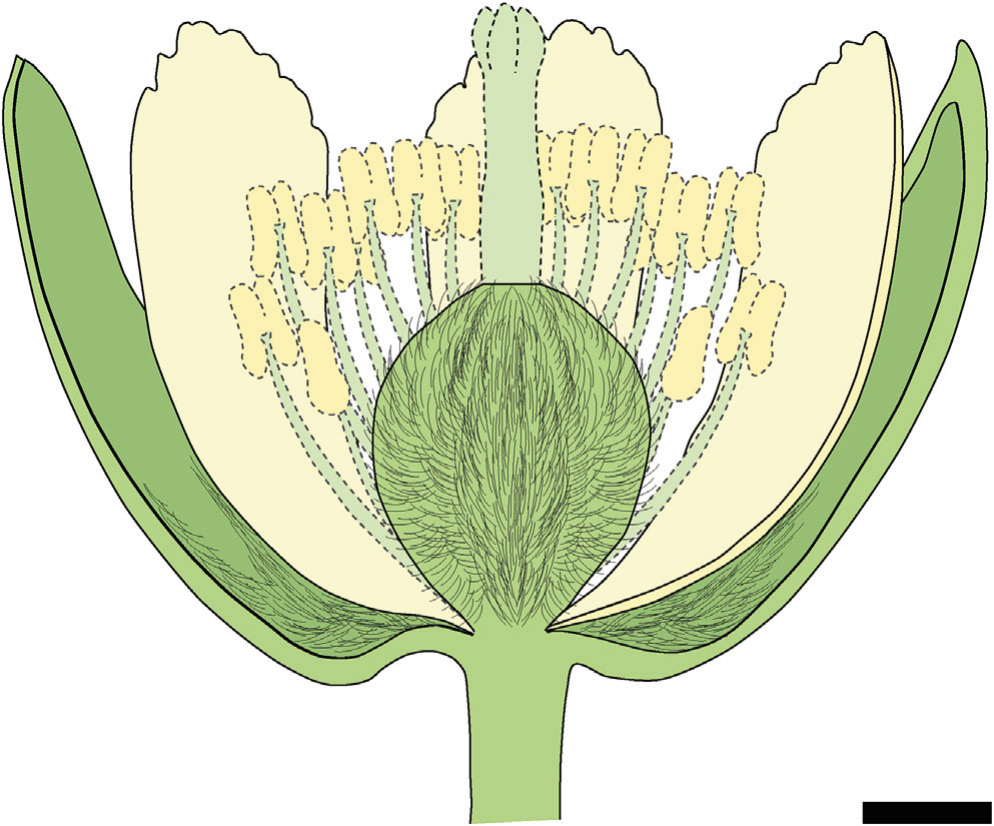
Artistic reconstruction of the flower morphology of *Tilia magnasepala* sp. nov. of the Oligocene of Enspel. Dashed lines indicate organs that were not fully preserved but inferred by comparison with extant species of *Tilia*. Bar, 1 mm.

*Remarks on description*: The flowers of *T. magnasepala* from Enspel correspond in general morphology to extant flowers of *Tilia* (Pigott, 2012; Table S1). Some missing characters include the shape of petals, the precise number of stamens, the presence or absence of staminodes and their morphology, as well as some morphological features of style and stigma. These missing characters are indicated by dotted lines in the artistic reconstruction of the flower (Fig. 4). All flowers/buds possess the same type of *in situ*/adhering *Tilia* pollen (Figs 3, S1–S4). The pollen corresponds to dispersed fossil pollen of *Intratriporopollenites instructus* (R.Potonié, 1931) P.W.Thomson & Pflug, 1953. Extant pollen of *Tilia*, and that of the genera *Craigia* and *Mortoniodendron* within the Tilioideae, have been investigated intensively with LM, SEM, and TEM (*e.g*. Zhang & Chen, 1984; Beug, 2004; Perveen *et al*., 2004; Sam & Auer, 2021; Sam *et al.,* 2024; Stebler, 2024; Geier *et al*., 2024a, 2024b, 2024c, 2024f, 2025; Halbritter *et al*., 2024a, 2024b) and as summarized by Geier *et al*. (2025). *Mortoniodendron* pollen is small‐ to medium‐sized, has a coarse reticulum, free‐standing columellae in the lumina and the muri are crested. Additionally, this genus exhibits an internal tectum in its pollen wall (Geier *et al*., 2025). Therefore, *Mortoniodendron* is excluded as a potential modern relative for our fossil. *Craigia* has pollen similar to *Tilia* but is unique in several features: Pollen is medium‐sized, reticulate to nanoreticulate in SEM, and has thickened costae at the apertures. The costae in *Craigia* pollen are 1.5–6.8 μm thick and 5.3–8.7 μm wide, vs 2.1–7 μm thick and 8.7–16 μm wide in *Tilia*. Pollen of *Tilia magnasepala* has costae 2.1–3.8 thick and 8–9 wide, conforming to both *Craigia* and *Tilia*. When the ultrastructure of the pollen wall is considered, *T. magnasepala* has no internal tectum (Fig. 3k), like extant *Tilia*, while recent and fossil *Craigia* pollen have an internal tectum (Zetter *et al*., 2002; Geier *et al*., 2025). Pollen from the fossil flowers of *T. magnasepala* has a distinct striate suprasculpture observed with SEM that corresponds to pollen from several extant species of *Tilia*, *for example T. americana*, *T. endochrysea*, *T. miqueliana*, and *T. tomentosa* (Perveen *et al*., 2004; Auer *et al*., 2024; Geier *et al*., 2024d, 2024e; Table S2). Similar dispersed fossil pollen grains have been documented with combined LM and SEM from various European Neogene localities, among others, the Miocene of Iceland (Denk *et al*., 2011), Germany (Vomela, 2016), Austria (Grímsson *et al*., 2020; Geier *et al*., 2022), and Türkiye (Bouchal, 2019).

*Comparison with flowers of extant* Tilia: Extant *Tilia* was investigated in detail by Pigott (2012), providing detailed descriptions and illustrations of whole plants, branches, leaves, buds, flowers, cuticles, and trichomes (Table S1). Based on the combined morphological features of all extant species of *Tilia*, the ‘general’ flower of the genus can be described as: bisexual, pentamerous, actinomorphic, with perianth differentiated into calyx and corolla, sepals sturdy, and petals rather delicate (Pigott, 2012). Trichomes are present on many flower parts (*e.g*. pedicels, sepals, ovaries, and/or styles) and can be simple, fasciculate (simple trichomes in bundles of two or more), or stellate with a varying number of arms. The androecium is organized in two whorls. The outer whorl produces numerous fertile stamens (25–60) while the inner either consists of five infertile staminodes or five fertile stamens. Staminodes have an intermediate morphology between stamens and petals (van Heel, 1966). The syncarpous gynoecium consists of a five‐carpellate, superior ovary, a single style, and a five‐lobed stigma. Nectar is produced by trichomatous nectaries composed of multicellular, clavate, glandular hairs located on the adaxial surface of the basal parts of the sepals (Vogel, 2000; Pigott, 2012; Konarska, 2013).

Flowers of *T. magnasepala* share numerous features with extant flowers of extant *Tilia*, such as the actinomorphic, pentamerous floral structure, the trichomatous nectaries, and the tilioid‐type pollen. Today's flowers of *Tilia* can be classified as bowl‐shaped or cup‐shaped depending on whether the perianth opens fully to a 180° angle or stays at an acute angle with inwardly curved petals and sepals. Access to nectar is open, and the flowers function as ‘revolver flowers’ during pollination: the visiting insect must circle around inside or on top of the flower sucking from one nectar cushion after the other and thereby dislodging pollen and providing pollination service (Vogel, 2000). *Tilia magnasepala* has an intermediate form based on the few specimens available; two are cup‐shaped (Fig. 2b,c), and one is bowl‐shaped (Fig. 2a), and their size range falls within the variation of extant *Tilia*. The sepal morphology of the fossils, ovate to triangular (Fig. 2a,d), is common in living species of *Tilia* (Table S1). Also, at the adaxial base of sepals there are simple trichomes, occurring individually or in pairs, supposedly forming trichomatous nectaries (Fig. 2e). Such trichomes at the adaxial base of the sepals are common in many living species of *Tilia* (Konarska, 2013) and other Malvaceae. In addition to the trichomes forming the nectaries, the sepal margins are covered in hairs which aid in the adherence of the sepals. The aestivation in *Tilia* is valvate, and the trichomes interlock and keep the buds closed until anthesis (Bayer & Kubitzki, 2003). On the contrary, the abaxial side of sepals usually has stellate trichomes in extant taxa (Pigott, 2012). The presence or absence of such trichomes on the abaxial side of sepals on the fossils of *Tilia* cannot be determined due to limitations of preservation. Nonetheless, trichomes covering the inside of the sepals are described for several extant species (*e.g*. Pigott, 2006, 2012; Table S1; Fig. 2j), especially for *T. cordata* (Konarska, 2013). Only remnants of the petals are preserved in the fossils, but they fall within the morphological range of extant floral dimensions for *Tilia* (Table S1). Regarding the stamens, little is left in *T. magnasepala*. The number of stamens in the fossil can only be estimated based on the remnants of the three stamens partly preserved and, based on symmetry, is suggested to have been at least five, which is in accordance with extant *Tilia* (Table S1). In two specimens of *T. magnasepala*, the spherical ovary is preserved (Fig. 2a,c) and is comparable to ovaries in some extant species of *Tilia* (Table S1). Finally, the fossil specimens have a dense indumentum of simple trichomes that cover the whole ovary (Fig. 2h). Five longitudinal lines can be observed on the ovary and represent either the carpel delimitation or the median plane (dorsal vascular bundle) of individual carpels. The ovaries of extant flowers of *Tilia* are generally also covered with trichomes; simple or fasciculate hairs can be found in several extant species of *Tilia* (Table S1).

Combining the morphological traits discussed above, it becomes apparent that all extant species of *Tilia* share characters with the fossils, but some more than others. Based on comparisons between these character states, two species share four out of five qualitative floral characters with *T. magnasepala* (Table S1). These species are *T. dasystyla* Steven and *T. miqueliana* Maxim., belonging to sect. Anastraea V. Engler and sect. Astrophilyra V. Engler, respectively (Pigott, 2012). Other species with similar floral traits are *T. endochrysea* Hand.‐Mazz. (sect. Endochrysea Chang H.‐t.), *T. japonica* (Miq.) Simonk. (sect. Anastraea), *T. mandshurica* Rupr. & Maxim., and *T. maximowicziana* Shiras. (both in sect. Astrophilyra). *Tilia magnasepala* shares typical floral morphology with extant *Tilia*, as reconstructed here (Fig. 4). However, based on the floral morphology of *T. magnasepala*, it is hard to assign it to a specific section or a current geographic group, since species producing flowers of similar morphology occur throughout the Northern Hemisphere. Regardless, the most defining feature of this flower, which also inspired its name, is the relatively large sepals, potentially subequal to the petals.

*Comparison with other fossil in situ Malvaceae pollen*: Fossil malvacean flowers with *in situ* pollen are rare, and only a handful have been investigated in depth (Table S3; Notes S1). Flowers of *Florissantia speirii* and *F. ashwillii*, from the Eocene to Oligocene of North America, contain reticulate, brevicolporate pollen with three to four apertures (Manchester, 1992). The *Florissantia* pollen type shows characteristic nonechinate malvaceous morphology, which is common in members of the subfamilies Sterculioideae, Bombacoideae, and Tilioideae. The distinctive features of *Florissantia* pollen grains are their psilate reticulum (vs striate in *T. magnasepala*) with lumina that decrease in size from the poles toward the equator (*F*. *speirii* vs uniform in *T. magnasepala*), their smaller size, and a less pronounced thickening (costae) around the apertures (*F. ashwillii*). *Craigia*, the sister genus to *Tilia*, now endemic to eastern Asia, is well represented by fossil fruits from the Eocene to Miocene of North America, Europe, and Asia (Kvaček *et al*., 2004), as well as *Craigia bronnii* flowers and buds with *in situ* pollen from the Neogene of Europe (Kvaček *et al*., 2002; Zetter *et al*., 2002; Geier *et al*., 2025; Table S3). Fossil pollen from *C. bronnii* flowers/buds is similar to that of *T. magnasepala*. However, the exine ornamentation in *C. bronnii* pollen is much finer than in *T. magnasepala* pollen, and only when observed in SEM can it be described as microreticulate. Additionally, the *C. bronnii* pollen grains tend to be smaller than those of *T. magnasepala*, and the horse‐shoe‐like thickening of the nexine around the apertures is more convex in *C. bronnii* than in *T. magnasepala*. Additionally, recent *Craigia* and fossil *C. bronnii* pollen have an internal tectum, an additional thin tectal layer embedded within the infratectum, which is lacking in extant *Tilia* and *T. magnasepala* pollen (Grímsson *et al*., 2020; Geier *et al*., 2025). Only two alleged *Tilia* flowers have been documented before this study (Table S3). Flowers of *T. parvulifolia* H.V. Smith from the late Eocene/early Oligocene of the United States, although associated with convincing fruiting bracts of *Tilia*, were never investigated for *in situ* pollen (Hall & Swain, 1971), so it cannot be compared with our material. Also, pollen extracted from a bundle of stamens from the Oligocene of England was investigated with LM only, and the pollen morphology was used to assign the stamens to *Tilia* (Chandler, 1957). The quality of the LM micrographs (Chandler, 1957, plate 15 fig. 130–132), however, is insufficient to confirm taxonomic assignment to *Tilia*. A reinvestigation with SEM and TEM would bring clarity and confirm or reject the taxonomic position of these stamens.

Kingdom Animalia Linnaeus, 1758

Phylum Arthropoda Gravenhorst, 1843

Class Insecta Linnaeus, 1758

Order Hymenoptera Linnaeus, 1758

Family Apidae Latreille, 1802

Subfamily Apinae Latreille, 1802

Clade Corbiculata Engel, 1998

Tribe Bombini Latreille, 1802

Genus *Bombus* Latreille, 1802, *s.l*.

Refer to Notes S2 for complete taxonomic descriptions and comments on the affinities of these species. In the following accounts, the morphological terminology follows that of Engel (2001) and Michener (2007). Nomenclatural acts are registered with ZooBank (www.zoobank.org), official registry of zoological nomenclature, with LSID: urn:lsid:zoobank.org:pub:D3D85866‐CCF6‐4D64‐BBCE‐8807A10BD98B.

*Kronobombus* Engel, subgen. nov.

*Type species*: *Bombus* (*Kronobombus*) *messegus* Engel et Wappler, sp. nov.

*Diagnosis*: Forewing with prestigma as long as pterostigmal width; depth of marginal cell relative to submarginal cells broad, marginal tangent not crossed by distance of 1rs‐m length within posterior of cell; 2 M notably angled posteriorly relative to Rs + M; 2Rs bowed; 2rs‐m strongly oblique, not sinuate, straight in anterior half; posterior portion of third submarginal cell extended apically at posterior arch; 2 m‐cu long, weakly curved; anterior border of second medial cell peaked. Hind wing with 2 M + Cu elongate, *c*.1.75× length of 1 M; distal abscissa of M absent; jugal lobe absent.

*Etymology*: The new subgeneric name is a combination of Ancient Greek *χρόνος* (*khrónos*, meaning, ‘time’) and *Bombus* (itself taken from *βόμβος*/*bómbos*, meaning, ‘buzzing’). The name is an allusion to the antiquity of the subgenus. The gender of the name is masculine.

ZooBank LSID: urn:lsid:zoobank.org:act:F16890FE‐E017‐4075‐8968‐CD7AFB2BBFF4.

**Fig. 5:**
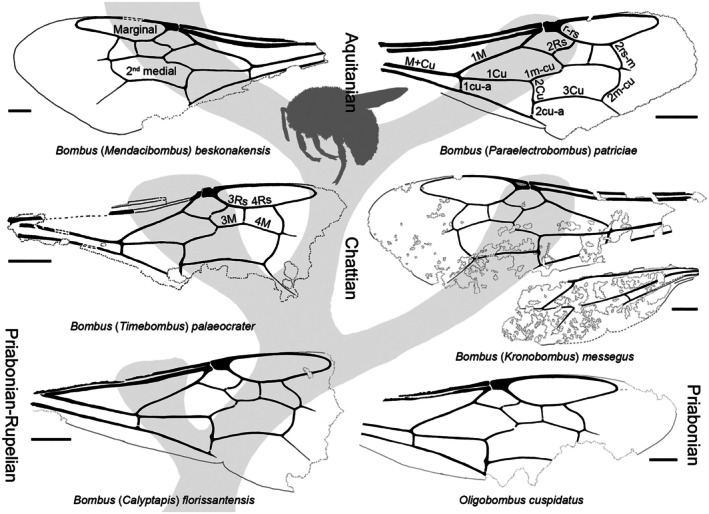
Wing venation in Eocene to Miocene bumble bees (*Bombus* Latreille) and their hypothetical relationships (refer to Systematic Paleontology and Supporting Information Notes S2). Important vein abscissae and cells labeled. Bars, 1 mm.

*Remarks on diagnosis*: This subgenus is seemingly an early‐diverging lineage of *Bombus* but perhaps not as much as *Calyptapis*, although both subgenera have the characteristic bombine bowed 2Rs (Fig. 5). Like *Calyptapis*, the depth of the marginal cell relative to the submarginal cells is broad, such that the marginal tangent (refer to Fig. S5a,b) is not crossed by the distance of 1rs‐m within the posterior of the cell (Fig. S5b; in *Timebombus*, *Paraelectrobombus* Nel & Petrulevičius, and extant subgenera the tangent is crossed by the length of 1rs‐m; the marginal cells of tribe Electrobombini, inclusive of *Oligobombus*, are like those of *Calyptapis* and *Kronobombus*: Fig. 5). It also retains a long and weakly curved 2 m‐cu and ‘peaked’ anterior border to the second medial cell (anterior border either continuous across 3 M and 4 M or weakly angled in *Timebombus*, *Paraelectrobombus*, and is typical for extant subgenera; Fig. 5). Like *Calyptapis*, 2rs‐m is strongly oblique (Fig. 5), with the posterior portion of the third submarginal cell extended apically at the posterior arch, but unlike *Calyptapis*, 2rs‐m is not sinuate and instead straight in its anterior half (Fig. 5). In addition, the prestigma is as long as the pterostigmal width and 2 M is notably angled posteriorly relative to Rs + M in *Kronobombus*, while in *Calyptapis*, the prestigma is distinctly shorter than pterostigmal width and 2 M is only weakly angled posteriorly relative to Rs + M (Fig. 5). Quite interestingly, the hind wing of *Kronobombus* lacks a jugal lobe as well as a distal abscissa to M (= indica vein of Apini), distinctive character states of *Bombus*, while 2 M + Cu is notably elongate, being *c*. 1.75× the length of 1 M (Fig. 5; in this regard similar, putatively symplesiomorpically, to the hind wing of *Electrobombus samlandensis*: *vide* Engel, 2001). As is typical for *Bombus*, the mandible retains outer mandibular grooves, is broadly curved apically, and the labrum is transverse.

*Bombus* (*Kronobombus*) *messegus* Engel et Wappler, sp. nov. (Figs 5, 6a–f, S5a,b, S6–S11).

**Fig. 6:**
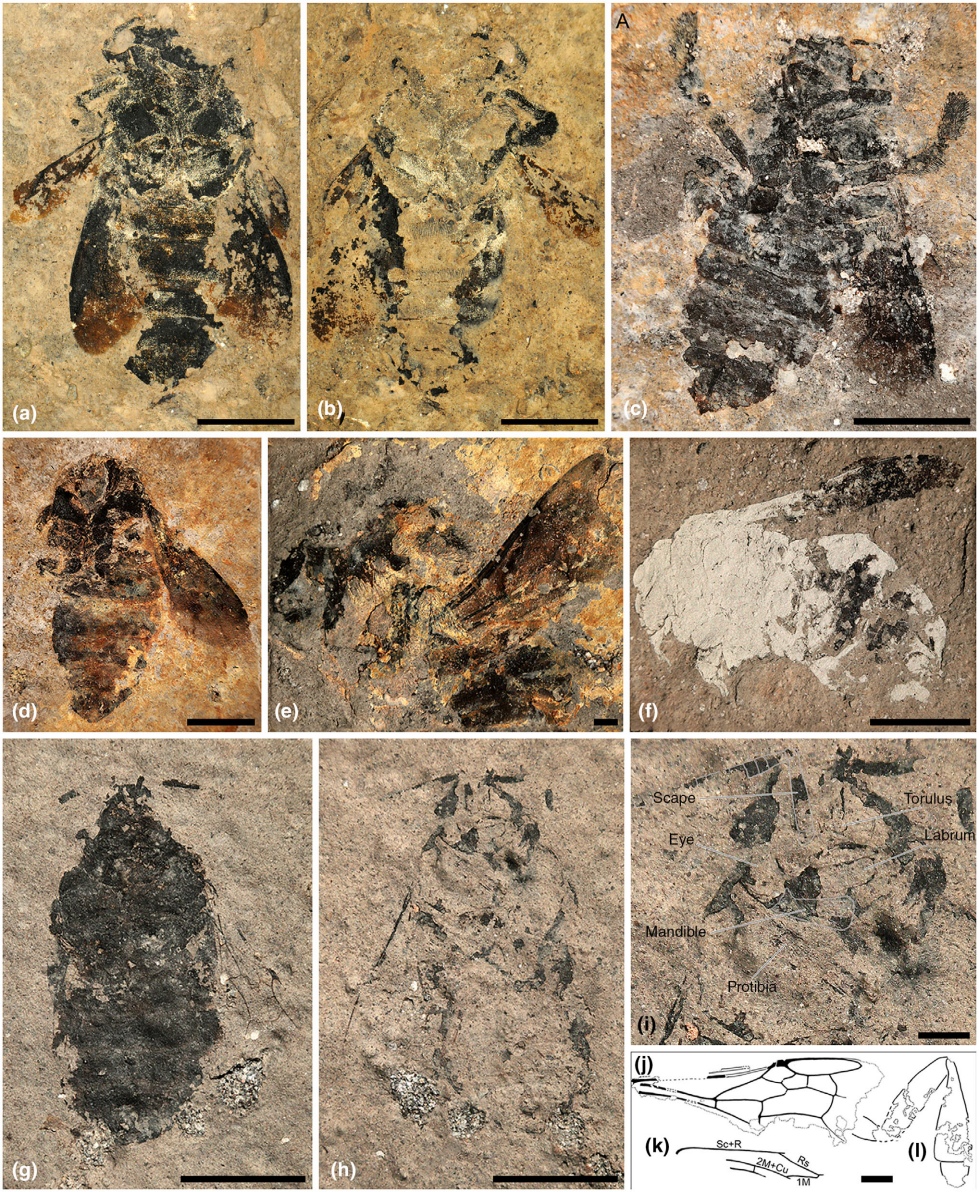
Fossil bees of the genus *Bombus* from the Oligocene of Enspel. (**a, b**) *Bombus* (*Kronobombus*) *messegus* subgen. et sp. nov., holotype female, NHMMZ PE 2001/5215‐LS. (**a**) Part. (**b**) Counterpart. (**c–f**) *Bombus* (*Kronobombus*) *messegus* subgen. et sp. nov., paratype and attributed females. (**c**) NHMMZ PE 1995/5321‐LS (paratype). (**d**) NHMMZ PE 1995/5243a‐LS (paratype; part; counterpart not depicted). (**e**) NHMMZ PE 1995/5314‐LS (paratype). (**f**) NHMMZ PE 1995/8792‐LS (attributed (nontype) female). (**g–l**) *Bombus* (*Timebombus*) *palaeocrater* subgen. et sp. nov., holotype female, NHMMZ PE 1997/6137‐LS. (**g**) Part. (**h**) Counterpart. (**i**) Detail of the head on counterpart with overlain drawings. (**j–l**) Wing venation and hindleg anatomy drawing, (**j**) = forewing, (**k**) = hindwing venation detail, (**l**) = hindleg. Bars, 5 mm (**a–h**), 1 mm (**i–l**).

*Holotype*: NHMMZ PE 2001/5215a‐LS (Fig. 6a) and NHMMZ PE 2001/5215b‐LS (Fig. 6b; part and counterpart).

*Paratypes*: NHMMZ PE 1995/5321‐LS (Fig. 6c), NHMMZ PE 1995/5243a‐LS (Fig. 6d) and NHMMZ PE 1995/5243b‐LS (part and counterpart), NHMMZ PE 1995/5314‐LS (Fig. 6e).

*Additional material*: One specimen is attributed to this species but is excluded from the type series owing to its general state of preservation, NHMMZ PE 1995/8792‐LS (Fig. 6f).

*Type locality*: Enspel, Rhineland‐Palatinate, Germany.

*Type stratum*: Enspel Fm, Layer S14; digging site G19B (Schindler & Wuttke, 2015).

*Age*: 24.56 ± 0.04 Ma, Chattian, Oligocene (Mertz *et al*., 2007).

*Etymology*: The specific epithet is the Ancient Greek adverb *μεσσηγύς* (*messēgús*, meaning, ‘in the middle’), referring to the putative intermediary position of the fossil between older stem‐groups and crown‐group *Bombus*.

ZooBank LSID: urn:lsid:zoobank.org:act:D8022166‐6FAB‐4CB9‐92C8‐2AFFEFE8D129.

*Diagnosis*: As for the subgenus (*vide supra*).

*Description*: ♀: Total body length (as preserved) 22.4 mm (paratype – PE 1995/5243: 20.7 mm, PE 1995/5314: 13.2 mm, PE 1995/5321: 18.9 mm), forewing length (as preserved) *c*. 13.6 mm (paratype PE 1995/5243: *c*. 14 mm, PE 1995/5314: *c*. 12 mm, PE 1995/5321: *c*. 9.5 mm). Integumental sculpture and coloration not preserved (integument represented by taphonomically altered carbonaceous compression; bee's integument likely black in life); body robust with abundant shaggy setae (coloration as preserved dark brown to black, whether colour patterns were present in life cannot be determined). Head broader than long (only observable obliquely in paratype PE 1995/5243a); labrum transverse (surface details not discernible); malar space apparently shorter than wide (based on paratype PE 1995/5243a); mandible with apical margin curved (not oblique) (paratype PE 1995/5321, apex of mandible in holotype missing), with outer mandibular grooves present (holotype and paratype PE 1995/5321). Mesosoma robust, much broader than head, maximum width 9 mm as preserved (paratype PE 1995/5243: *c*. 8 mm, PE 1995/5321: *c*. 6 mm), fringe of setae particularly elongate on mesoscutellum (observable in paratype PE 1995/5314). Legs with abundant setae; metatibia comparatively narrow, about as long as metatrochanter + metafemur, with fringes of setae bordering corbicular surface, with profundal setae (seemingly over proximal third) (most easily visible in paratype PE 1995/5243a); metabasitarsus longer than wide, length *c*. 1.5× apical width, about as broad as metatibia apically, margins not converging apically, apical margin straight, apical angle rounded, margins with fringes of short bristles, without fringe of elongate setae (such fringe present in *Mendacibombus* Skorikov) (most easily visible in paratype PE 1995/5243a).

Forewing with membrane darkly infumate, lighter apically beyond closed cells; 1 M slightly proximal to 1cu‐a, straight; 1Rs short, shorter than Rs + M; Rs + M slightly longer than 2 M; 2Rs bowed anteriorly; pterostigma slightly longer than wide, prestigma as long as pterostigmal width; marginal cell broad, apically slightly offset from anterior wing margin, apex narrowly rounded, not appendiculate, free portion of marginal cell shorter than portion bordering submarginal cells; r‐rs slightly longer than 3Rs; 4Rs longer than 3Rs; 1 m‐cu entering second submarginal cell slightly proximal midlength, thus 2 M slightly shorter than 3 M; 3 M angled at juncture with 4 M (*i.e*. second medial cell ‘peaked’), shorter than 4 M; 1rs‐m straight; 2rs‐m oblique, straight anteriorly, gently arched posteriorly; 4Rs shorter than 4 M, slightly less than 0.75× 4 M; third submarginal cell longer than either first or second submarginal cells, only slightly longer than the second submarginal cell; 2 m‐cu not oblique, weakly curved along length, proximal 2rs‐m by 4× vein width, nearly twice as long as 2Cu; 3Cu in medial position along 2Cu/2cu‐a. Hind wing with short distal abscissa of Rs, longer than rs‐m; without distal abscissa of M; 2 M + Cu elongate, 1.75× 1 M; jugal lobe absent. Metasoma robust, maximum length as preserved 11 mm (paratype PE 1995/5243: 12 mm, PE 1995/5321: 10 mm); sting long, longer than metasomal sternum VI (visible in holotype and paratypes PE 1995/5321 and PE 1995/5243a: Fig. 6a,c,d), valvulae of equal widths, thickened proximally at attachment to rami, rami thin, simple.

♂: *Latet*.

*Timebombus* Engel, subgen. nov.

*Type species*: *Bombus* (*Timebombus*) *palaeocrater* Engel et Wappler, sp. nov.

*Diagnosis*: Malar space short, broader than long; labrum with faint medial furrow, broader basally and thin apically; flagellomere I as long as combined lengths of flagellomeres II and III. Forewing with prestigma about as long as pterostigmal width; depth of marginal cell relative to submarginal cells narrow, marginal tangent crossed by distance of 1rs‐m length within posterior of cell; 2 M notably angled posteriorly relative to Rs + M; 2Rs bowed; 2rs‐m slightly oblique, straight anteriorly, weakly arched posteriorly, not prominently extended apically; 2 m‐cu long, weakly curved; anterior border of second medial cell peaked. Hind wing with 2 M + Cu slightly longer than 1 M; distal abscissa of M absent.

ZooBank LSID: urn:lsid:zoobank.org:act:F28E4B28‐1CFB‐4BE0‐853B‐46491F684BDE.

*Remarks on diagnosis*: Quite unlike *Calyptapis* and *Kronobombus*, the species of this subgenus has a narrow marginal cell, like that of extant lineages of *Bombus*. A further derived feature is the slightly oblique 2rs‐m, with the anterior border of the third submarginal cell closer in length to the posterior border, and with 2rs‐m straight anteriorly and weakly arched posteriorly without a prominent extension apically. It also has the bombine bowed 2Rs indicative of all *Bombus s.l*. Unlike more derived bombines, however, 2 m‐cu is long and weakly arched, rather than oblique and posteriorly extending more proximally as is the case in most extant *Bombus* and in the subgenus *Paraelectrobombus*. Unlike *Paraelectrobombus*, the second medial cell is ‘peaked’ rather than 3 M and 4 M rather continuous, without an angle at their juncture. In extant subgenera of *Bombus*, the second medial cell is about as broad apically (2 m‐cu) as it is proximally where it borders 2Cu, while in *Timebombus*, *Paraelectrobombus*, *Kronobombus*, and *Calyptapis* the second medial cell is distinctly broader apically. The hind wing is partly visible in the part of the holotype, with Sc + R particularly clear near the wing base and becoming fainter to trace as it extends apically; Rs, rs‐m, 1 M, M + Cu, cu‐a, and A are faint but can be traced by following the margins of the tubular veins, although 1 M + Cu and 1A are not complete as extending toward the base. Although incomplete and faint, it is noteworthy that the distal abscissa of M is absent and 2 M + Cu is only slightly longer than 1 M (Fig. 6k), the former consistent with all Bombini and the latter a feature similar to extant subgenera of *Bombus* (the membrane of the hind wing is not discernible). The malar space is apparently short, broader than long; the labrum with a faint medial furrow, broader basally and thin apically; and flagellomere I is as long as the combined lengths of flagellomeres II and III (Fig. 6i; much like in *Mendacibombus*).

*Etymology*: The new genus‐group name is a euphonious combination of the Middle English *time* (identical to the Modern English word) and the generic name *Bombus*. The name refers to the antiquity of the subgenus. The gender of the name is masculine.

*Bombus* (*Timebombus*) *palaeocrater* Engel et Wappler, sp. nov. (Figs 5, 6g–l, S5c,d, S12)

*Holotype*: NHMMZ PE PE1997/6137a‐LS (Fig. 6g) and NHMMZ PE 1997/6137b‐LS (Fig. 6h,i; part and counterpart).

*Type locality*: Enspel, Rhineland‐Palatinate, Germany.

*Type stratum*: Enspel Fm, Layer S16; digging site G10 (Schindler & Wuttke, 2015).

*Age*: 24.56 ± 0.04 Ma, Chattian, Oligocene (Mertz *et al*., 2007).

*Etymology*: The specific epithet is a combination of the Ancient Greek adjective *πᾰλαιός* (*palaiós*, meaning, ‘ancient’) and the noun *κρᾱτήρ* (*krātḗr*, meaning, ‘crater’, ‘mouth of a volcano’), and refers to the crater lake in which the fossil was deposited.

ZooBank LSID: urn:lsid:zoobank.org:act:6F9C987C‐ECD6‐4498‐AC28‐8127A96F9177.

*Diagnosis*: As for the subgenus (*vide supra*).

*Description*: ♀: Total body length (as preserved) 15 mm, forewing length (as preserved) *c*. 7.7 mm. Integument sculpture and coloration not preserved (integument is represented by carbonaceous compression taphonomically altered; the bee's integument was likely black in life (like most bumble bees) but the dark coloration of preserved setae may or may not reflect an overall dark pubescence throughout the body); body robust with abundant setae (where evident) (likely abundantly pubescent in life as in modern bumble bees). Head apparently about as long as wide (as preserved), distinctly narrower than mesosoma (note that the head is slightly oblique as preserved, with the vertex tilted slightly backward, such that length of head is slightly foreshortened as seen, but degree to which the head may have been longer than wide is uncertain but if longer; then, it would have been scarcely so; perhaps of proportions similar to species like *Bombus* (*Cullumanobombus*) *rufocinctus* Cresson or at most like medium‐length bumble bees). Antennal toruli apparently at head midlength, separated by a distance equivalent to torulus diameter; scape long, surpassing vertex; flagellomere I long, about as long as combined lengths of flagellomeres II and III (similar in this respect to *Mendacibombus*); clypeal furrow distinct, broad, nearly 0.2× labral length; labrum transverse, with faint medial furrow (likely shallow and weak in life), wide along proximal margin but quickly narrowing and thin apically; malar space apparently shorter than wide; mandible with apical margin curved (not oblique), with outer mandibular grooves present. Mesosoma quite robust, maximum width as preserved 6.7 mm (propleura easily discernible in part). Metatibia about as long as metatrochanter + metafemur; metatibia with corbicula (given that individual was obviously foraging the bee was likely a worker); mesosoma nearly as long as metasoma (as preserved). Forewing (Figs 6j, S5c) with membrane apparently hyaline, clear (Fig. 6g); 1 M slightly proximal to 1cu‐a, straight; 1Rs short, shorter than Rs + M; Rs + M longer than 2 M; 2Rs bowed anteriorly; pterostigma slightly longer than wide, prestigma about as long as pterostigmal width; marginal cell narrow, apically slightly offset from anterior wing margin, apex narrowly rounded, with minute appendiculate stub, the free portion of the marginal cell shorter than the portion bordering submarginal cells; r‐rs about as long as 3Rs; 4Rs much longer than 3Rs; 1 m‐cu entering the second submarginal cell in proximal third, thus 2 M distinctly shorter than 3 M; 3 M angled at juncture with 4 M (*i.e*. second medial cell ‘peaked’), shorter than 4 M; 1rs‐m straight; 2rs‐m slightly oblique, straight anteriorly, weakly arched posteriorly, thus the third submarginal cell only slightly extended apically in the posterior section, 4Rs shorter than 4 M, slightly more than 0.76× 4 M; the third submarginal cell longer than either the first or second submarginal cells; 2 m‐cu not oblique, weakly curved along length, proximal 2rs‐m by 2× vein width, much longer than 2Cu; 3Cu in medial position along 2Cu/2cu‐a. Hind wing (Fig. 6k) with short distal abscissa of Rs, about as long as rs‐m; 2 M + Cu only slightly longer than 1 M (hind wing largely missing except Sc + R extending from base to divergence of Rs, Rs extending to rs‐m, with short distal abscissa Rs and no distal abscissa M, small portion of 1 M present faintly and extending proximal for distance about equivalent to rs‐m, then effaced in fossil).

♂: *Latet*.

*Remarks*: The bee is preserved lengthwise on its back, with the legs either tucked in under the body (forelegs and midlegs) or tightly alongside the body (hindlegs). The bee's left wings are folded back over the body and therefore difficult to discern in the part and entirely obscured in the counterpart; the right forewing and hindwing are extended slightly obliquely away from the body, making them more visible, at least in the part. The head is slightly tilted, thereby appearing slightly foreshortened but nicely exposing the mandibles, labrum, and from an oblique slant the clypeus, exposing its apical furrow above the labrum. The outlines of head and foreleg structures are nicely preserved in the counterpart. There is more integument preserved in the part, but given how black and taphonomically altered they are, many details of the head and mesosoma are less easily observed. The part appears to show the ental (*i.e*. the internal surface of the sclerite that faces the interior of the body) surfaces of structures seen in ventral view in the counterpart.

## Discussion

### Past and present pollination biology of *Tilia*

All six fossil bumble bee specimens have an abundance of *Tilia* pollen grains adhering to their exoskeleton (Figs S6–S12). The mesosomas and legs yielded the most pollen (Figs S6d–f, S8h, S10h). As for the head, we detected pollen both ventrally between mouthparts and the compound eyes, as well as distally in the space between the head and the propleura (Figs S6c, S8c, S9d, S10c, S12c), the latter of which would correspond to pollen associated with the extended mouthparts. Pollen was also found amidst the metasomal sterna (Fig. S10m). One leg of *B. messegus* was completely packed with *Tilia* pollen (Fig. S10t). Generally, the bees' ventral surfaces were covered with more pollen than the ‘dorsal surfaces’. The amount of pollen found ventrally is not surprising given that flowers of *Tilia* (recent and the fossil) are bowl‐shaped and robust bees landing on them must crawl across the perianth to drink nectar from nectaries at the base of the sepals. As with living bumble bees, the fossil Oligocene bumble bees must have actively moved through the flowers of *Tilia magnasepala*. During their movement, the abundant villous setae brushed over the *T. magnasepala* stamens and pollen from anthers was dislodged and adhered to the mesosomas of the bumble bees. Based on the position and concentration of pollen loads on the fossil bumble bees, it appears that they also actively collected *T. magnasepala* pollen by scraping over the stamen surfaces with their forelegs, as evident by pollen entrapped amid setal branches. Ultimately, the bees would have groomed themselves, mixing pollen with a bit of regurgitated nectar, and then moved the pollen from forelegs and mesosoma to the midlegs and hindlegs. In spite of grooming, the fossil bees still have significant pollen remaining on their heads, mesosomal venters and sides, and parts of their metasomas. As a last step, they would have rubbed the metabasitarsal ctenidia, which transfers pollen proximally to the pollen press and compresses, as the metatibial‐metabasitarsal joint is flexed, the pollen mixture into the apex of the corbicula. Repeating this process makes the pollen pack progressively larger as more and more pollen is pushed into the corbicula, eventually producing a rather sizeable pollen pellet for transport back to the nest. However, we did not encounter any full‐sized pollen pellets (*i.e*. ambrosia) on the fossil bees. Some bees did not preserve the corbiculae, and even those who did lacked pellets. Since extant pollen pellets dissolve easily in water, this might account for the lack of ambrosia on the fossil bees.

Present‐day pollination biology, floral visitation, and floral resources of at least 13 species of *Tilia* have been investigated across the distribution range of the genus (United States, Europe, South Korea; Anderson, 1976; Corbet *et al*., 1979; Chung & Kim, 1984; Pawlikowski, 2010; Pigott, 2012; Koch & Stevenson, 2017; Jacquemart *et al*., 2018), except for West Asia. Pollination by insects is the primary pollination mode. The four hyperdiverse insect orders – Coleoptera, Diptera, Lepidoptera, and Hymenoptera – have been observed visiting, foraging pollen and/or nectar, and pollinating flowers of *Tilia*. Beetles of the Buprestidae, Cerambycidae, Coccinellidae, and Scarabaeidae are known to visit *Tilia* flowers. However, the relatively smooth bodies of the beetles, ornamented with few comparatively sparse setae, often carry few pollen grains. Nonetheless, the relative concentration of pollen of *Tilia* on their bodies can be high (Anderson, 1976; Chung & Kim, 1984), suggesting that these beetles can be active pollinators for the genus. Flies, being more densely setose than most beetles, often carried more pollen than beetles adhering to their exterior. Flies of the families Agromyzidae, Empididae, Syrphidae, Sarcophagidae, Tabanidae, and Tachinidae are known to visit flowers of *Tilia* (Anderson, 1976; Corbet *et al*., 1979; Chung & Kim, 1984; Pigott, 2012; Jacquemart *et al*., 2018), and in one study, syrphid flies represented the majority of floral visitors, emphasizing their role in the pollination of modern *Tilia* (Pigott, 1991). Among the Lepidoptera, butterflies had no or few pollen grains adhering, while moths carried exclusively pollen of *Tilia* in varying amounts (Anderson, 1976), highlighting their role in the broader suite of pollinating insects. Nevertheless, the main group of present‐day floral visitors and pollinators for *Tilia* is hymenopterans, especially bees. The bodies of bees are often covered with *Tilia* pollen following visits, and they usually visit several flowers consecutively and are believed to contribute the greatest pollination service for this genus (Anderson, 1976; Corbet *et al*., 1979; Chung & Kim, 1984; Pawlikowski, 2010; Pigott, 2012; Koch & Stevenson, 2017; Jacquemart *et al*., 2018). Five families of bees occurring in the Northern Hemisphere have been reported visiting *Tilia* flowers (*i.e*. Andrenidae, Apidae, Colletidae, Halictidae, and Megachilidae). The bees were observed to pack mostly ‘pure’ pollen from *Tilia* on their corbiculae or scopae, or metasomal scopae in the case of megachilids. The most frequent visitor among bees was the cultivated honey bee, *Apis mellifera* L. However, among wild bees, bumble bees, *Bombus*, were the main floral visitors foraging on *Tilia*. Until now, at least 18 extant species of *Bombus* have been reported carrying large pollen pellets of *Tilia* in their corbiculae (Anderson, 1976; Corbet *et al*., 1979; Chung & Kim, 1984; Pawlikowski, 2010; Pigott, 2012; Koch & Stevenson, 2017; Jacquemart *et al*., 2018). Field observations by two authors (CG and FG) corroborate previous reports (Melville, 1949) that honey bees were observed collecting nectar rather than pollen (without ambrosia, *i.e*. pollen pellet) when visiting flowers of *Tilia*. By contrast, we also observed bumble bees collecting pollen and forming pollen pellets composed almost entirely of *Tilia* pollen (Fig. S13). Extant species of *Bombus* are generalist pollinators and therefore not usually specialized to a given plant genus or family but will usually visit large stands of different suitable flowers (Free, 1970; Leonhardt & Blüthgen, 2012; Yourstone *et al*., 2023). The same was likely true for the fossil species, with the adhering pollen likely representing their last floral visits before death and reflecting a general abundance of suitable and attractive floral patches of *Tilia*. Although none of the fossil bumble bees preserved corbiculae filled with pollen (several specimens did not preserve the corbiculae at all; as described in the previous section), the bees likely came in close contact with all parts of the flower, ended up covered in pollen, and most certainly transported pollen from one flower to another (Figs. S6–S12). Applying the definitions of Peña‐Kairath *et al*. (2023), based on the amount and position of fossil *Tilia* pollen on the bees, combined with the fact that extant species of *Bombus* visit and pollinate flowers of *Tilia*, we can conclude that the fossil bumble bees of Enspel were true pollinators of *Tilia*.

### Origin and biogeographic history of *Tilia* and *Bombus* and their spatiotemporal interaction

Dated phylogenies, incorporating fossils, suggest that Tilioideae diverged from the remaining Malvaceae *c*. 79 Ma and started to diversify *c*. 73 Ma. The lineage leading to present‐day *Mortoniodendron* diverged around the same time (*c*. 73 Ma). Shortly thereafter, *c*. 65 Ma, *Craigia* and *Tilia* diverged, followed by further diversification within *Tilia c*. 49 Ma (Hernández‐Gutiérrez & Magallón, 2019). Similarly, dated phylogenies for the origin of corbiculate bees (Apini, Bombini, Electrapini, Electrobombini, Euglossini, Melikertini, and Meliponini) are estimated between 87 and 70 Ma (Cardinal & Danforth, 2013), with subsequent diversification at 40 and 25 Ma leading to, among others, *Bombus* (Hines, 2008).

The earliest fossil records of both *Tilia* and *Bombus* date back to the Eocene of North America. The *Tilia* records either predate the first *Bombus* fossil by *c*. 15 million years or they are contemporaneous. The earliest *Tilia* macrofossils are leaves from the middle Eocene (Ypresian, *c*. 49 Ma; Wolfe *et al*., 2003) Republic flora, Washington, USA (Wolfe & Wehr, 1987). More reliable are infructescence bracts from the late Eocene (Priabonian, *c*. 38–34 Ma) Bull Run flora, Nevada, USA (Manchester, 1994). The earliest fossil *Bombus*, *Bombus* (*Calyptapis*) *florissantensis* (Cockerell, 1906), is from the late Eocene (Priabonian, *c*. 34 Ma; Evanoff *et al*., 2001) of Florissant, Colorado, USA (Dehon *et al*., 2019); *Tilia* pollen has also been reported from this locality, but see Bouchal *et al*. (2016) who found Bombacaoideae/Sterculioideae gen. indet. pollen. The earliest European records of *Tilia* (pollen: Zetter *et al*., 2024, pers. obs. FG) stem from the latest Eocene (Priabonian, *c*. 34 Ma; Hooker *et al*., 2009) Insect Limestone, Isle of Wight, UK. No true *Bombus* occurs at this locality, but the electrobombine *Oligobombus cuspidatus* Antropov (in Antropov *et al*., 2013) co‐occurs in the same deposits (refer to Supporting Information Notes S2 regarding affinities of *Oligobombus*). From mainland Europe, the oldest bracts of *Tilia*, accompanied by fossil leaves, are from the early Oligocene (Rupelian) of France and Czech Republic (Hably *et al*., 2000; Kvaček & Walther, 2004) followed by leaves assigned to *Tilia gigantea* Ettingshausen from the late Oligocene (Chattian; *c*. 24 Ma; Mertz *et al*., 2007) of Enspel, Germany (Köhler & Uhl, 2014; Tables S4–S8). This locality also includes the *Tilia magnasepala* flowers and *B*. (*Kronobombus*) *messegus* and *B*. (*Timebombus*) *palaeocrater* described herein. Interestingly, the sediments of Enspel have not yielded the persistent bracts unique to *Tilia*. From the Miocene onwards, *Tilia* is known from across Europe (Mai, 1961; Manchester, 1994; Krutzsch, 2004; Pigott, 2012; Stuchlik *et al*., 2014). At several of these localities, especially of Early Miocene age, *Tilia* and *Bombus* co‐occur in fossil assemblages. These include *B*. (*Mendacibombus*) *beskonakensis* (Nel & Petrulevicius, 2003) from the early Burdigalian (*c*. 19.7–17.9 Ma; Paicheler, 1978; Denk *et al*., 2017) of Güvem (Bes‐Konak) Anatolia, Türkiye, co‐occurring with infructescences of *Tilia* (Paicheler, 1978; Denk *et al*., 2017). *Bombus crassipes* (Novák, 1878) from the late Aquitanian to middle Burdigalian (*c*. 21–17 Ma; Rojík, 2004) of Mokřina (Krottensee), Czech Republic, co‐occurring with *Tilia* (Bůžek & Holý, 1996). *Bombus* (*Cullumanobombus*) *trophonius* (Prokop *et al*., 2017) from the early Burdigalian (*c*. 20–18 Ma; Rajchl *et al*., 2009) of Bílina, Czech Republic, is found alongside leaves and infructescences of *Tilia* (Kvaček *et al*., 2004). Younger records include *B*. (*Cullumanobombus*) *pristinus* (Unger, 1867; Dehon *et al*., 2019) from the Burdigalian (*c*. 20–16 Ma; Velitzelos *et al*., 2014) of Kimi, Euboea, Greece, along with infructescence of *Tilia knoblochii* (Velitzelos *et al*., 2005, 2014). In the Randeck Maar Lake, Germany, *B*. (*Cullumanobombus*) *randeckensis* Wappler & Engel (in Wappler *et al*., 2012) from the Langhian (17–15 Ma; Rasser *et al*., 2013) is reported together with *Tilia* pollen (Kottik, 2002; Rasser *et al*., 2013). The latest report of fossil *Bombus* is from Spain, *B*. (*Melanobombus*) *cerdanyensis* Dehon & Engel (in Dehon *et al*., 2014), from the Tortonian (*c*. 12–7 Ma; Agustí & Roca, 1987) of La Cerdanya, occurring with *Tilia* pollen (Jiménez‐Moreno *et al*., 2010).

As evident from the European fossil record, *Bombus* and *Tilia* co‐occurred over millions of years, a biogeographic relationship that extends to the present day. The reason for this co‐occurrence likely lies in the climatic preferences of both groups rather than a biological dependence on each other. Biologically, the two taxa are certainly important for each other (food source vs pollination service), but *Tilia* is known to be visited by up to 135 insect taxa (*e.g*. Anderson, 1976) and *Bombus* visits not only *Tilia* but a plethora of plant taxa for both nectar and/or pollen (*e.g*. Free, 1970; Yourstone *et al*., 2023). During the middle to late Eocene, the time period in which both *Tilia* and *Bombus* trace back their earliest occurrences (as described in the previous section), Earth experienced a global warm‐house climate (Zachos *et al*., 2001; Mosbrugger *et al*., 2005; Westerhold *et al*., 2020). This allowed *Tilia* to thrive farther north in North America and disperse across the North Atlantic Land Bridge, while *Bombus*, which prefer colder temperatures, were not limited by temperatures in the North and could have spread at any time when connections between continents were possible. The earliest‐diverging extant clades of *Bombus* are largely Holarctic or Palearctic (Hines, 2008) while the earliest occurrence for the genus is from the latest Eocene of central North America, but the direction of their dispersion (Americas to Eurasia vs Eurasia to the Americas) is unclear given the paucity of their fossil record, especially in Asia (Dehon *et al*., 2019). Regardless, at the Eocene–Oligocene transition, and during the Oligocene and especially the Miocene, both *Tilia* and *Bombus* were widespread across continental Europe. Today, *Bombus* thrive best in cold‐temperate rather than subtropical climates (Hines, 2008), which is within the climate and biome preferences of *Tilia* (Geier *et al*., 2025). This suggests that the long‐lasting relationship between *Tilia* and *Bombus* is driven by climatic factors rather than obligatory biological synergism.

## Competing interests

None declared.

## Author contributions

Conceptualization: CG and FG. Methodology; investigation; writing – review and editing: CG, MSE, JMB, SU, JS, DU, TW, SW, LB and FG. Data curation: CG, JMB, SU and FG. Writing – original draft: CG, MSE and FG. Supervision; project administration: FG. Funding acquisition: FG and SW. All authors read and approved the final manuscript.

## Disclaimer

The New Phytologist Foundation remains neutral with regard to jurisdictional claims in maps and in any institutional affiliations.

## Supporting information


**Fig. S1** Oligocene *Tilia magnasepala* C.Geier & Schönenb. flower bud from Enspel, Germany, NHMMZ PB 2013/5272.
**Fig. S2** Oligocene *in situ* pollen extracted from flowers of *Tilia magnasepala* C.Geier & Schönenb. sp. nov. from Enspel, Germany, LM and SEM.
**Fig. S3** Oligocene *in situ* pollen extracted from flowers of *Tilia magnasepala* C.Geier & Schönenb. sp. nov. from Enspel, Germany, detail SEM micrographs.
**Fig. S4** TEM micrographs of *in situ* pollen grains extracted from the flower of *Tilia magnasepala* C.Geier & Schönenb. sp. nov. NHMMZ PB 2017/5564‐LS of Enspel, unstained.
**Fig. S5** Wing venation of Late Oligocene bumble bees (*Bombus* Latreille) from Enspel.
**Fig. S6** *Bombus* (*Kronobombus*) *messegus* Engel & Wappler, sp. nov., NHMMZ PE 2001/5215 with adhering *Tilia* pollen (Fig. S7a–o).
**Fig. S7** Fossil *Tilia* pollen extracted from the exoskeleton of *Bombus* (*Kronobombus*) *messegus* Engel & Wappler, sp. nov. NHMMZ PE 2001/5215 (Fig. S6).
**Fig. S8** *Bombus* (*Kronobombus*) *messegus* Engel & Wappler, sp. nov., NHMMZ PE 1995/5243 with adhering *Tilia* pollen.
**Fig. S9** *Bombus* (*Kronobombus*) *messegus* Engel & Wappler, sp. nov., NHMMZ PE 1995/5314 with adhering *Tilia* pollen.
**Fig. S10** *Bombus* (*Kronobombus*) *messegus* Engel & Wappler, sp. nov., NHMMZ PE 1995/ 5321 with adhering *Tilia* pollen
**Fig. S11** *Bombus* (*Kronobombus*) *messegus* Engel & Wappler, sp. nov., NHMMZ PE 1995/8792 with adhering *Tilia* pollen.
**Fig. S12** *Bombus* (*Timebombus*) *palaeocrater* Engel & Wappler, sp. nov., NHMMZ PE 1997/6137 with adhering *Tilia* pollen.
**Fig. S13** Extant bumble bees caught on silver lime (*Tilia tomentosa*) at the Botanical Garden of the University of Vienna, June 2024.
**Notes S1** The fossil record of Malvaceae flowers, morphological comparison of *Tilia magnasepala* sp. nov. and the ecology of extant *Tilia*.
**Notes S2** Systematic Palaeontology on *Bombus* Latreille.

**Table S1** The flower morphology of recent and fossil *Tilia* flowers.
**Table S2** The pollen morphology and ultrastructure of recent and fossil *Tilia*.
**Table S3** The fossil record of Malvaceae flowers.
**Table S4** The macroflora of Enspel.
**Table S5** The microflora of Enspel.
**Table S6** The fossil flora of Enspel.
**Table S7** Systematic list of micro‐ and macrofloral elements from Enspel and their ecological characteristics.
**Table S8** Vegetation units of the Enspel paleoflora.
Please note: Wiley is not responsible for the content or functionality of any Supporting Information supplied by the authors. Any queries (other than missing material) should be directed to the *New Phytologist* Central Office.

## Data Availability

The fossil material described and illustrated herein is housed in the collections of the Naturhistorisches Museum Mainz/Landessammlung für Naturkunde Rheinland‐Pfalz, Mainz (NHMMZ, Germany) and can be accessed by contacting the collection manager. The accession nos. for the *Tilia* fossils are as follows: NHMMZ PB 2013/5272‐LS, NHMMZ PB 2014/5218‐LS, NHMMZ PB 2017/5055‐LS, and NHMMZ PB 2017/5564‐LS, and the accession nos. for the Bombus fossils are as follows: NHMMZ PE 1995/5243‐LS, NHMMZ PE 1995/5314‐LS, NHMMZ PE 1995/5321‐LS, NHMMZ PE 1995/8792‐LS, NHMMZ PE 2001/5215‐LS, and NHMMZ PE PE1997/6137‐LS. One herbarium specimen of *Tilia americana* has been photographed from the herbarium of the University of Vienna, accession no.: WU 0151993. All data generated or analyzed during this study are included in this published article and its Supporting Information files. For replicability, specifically refer to Figs 2, 3, 6, S1–S12; Tables S1, S2.
