# Supplementary material for: 24 million years of pollination interaction between European linden flowers and bumble bees
Source: New Phytol. 2025 Sep 22;248(4):2111–27. doi: 10.1111/nph.70531 (PMC12529026; doi:10.1111/nph.70531)
Supplement: Supplementary file 1 — Fig. S1 Oligocene Tilia magnasepala C.Geier & Schönenb. flower bud from Enspel, Germany, NHMMZ PB 2013/5272. Fig. S2 Oligocene in situ pollen extracted from flowers of Tilia magnasepala C.Geier & Schönenb. sp. nov. from Enspel, Germany, LM and SEM. Fig. S3 Oligocene in situ pollen extracted from flowers of Tilia magnasepala C.Geier & Schönenb. sp. nov. from Enspel, Germany, detail SEM micrographs. Fig. S4 TEM micrographs of in situ pollen grains extracted from the flower of Tilia magnasepala C.Geier & Schönenb. sp. nov. NHMMZ PB 2017/5564‐LS of Enspel, unstained. Fig. S5 Wing venation of Late Oligocene bumble bees (Bombus Latreille) from Enspel. Fig. S6 Bombus (Kronobombus) messegus Engel & Wappler, sp. nov., NHMMZ PE 2001/5215 with adhering Tilia pollen (Fig. S7a–o). Fig. S7 Fossil Tilia pollen extracted from the exoskeleton of Bombus (Kronobombus) messegus Engel & Wappler, sp. nov. NHMMZ PE 2001/5215 (Fig. S6). Fig. S8 Bombus (Kronobombus) messegus Engel & Wappler, sp. nov., NHMMZ PE 1995/5243 with adhering Tilia pollen. Fig. S9 Bombus (Kronobombus) messegus Engel & Wappler, sp. nov., NHMMZ PE 1995/5314 with adhering Tilia pollen. Fig. S10 Bombus (Kronobombus) messegus Engel & Wappler, sp. nov., NHMMZ PE 1995/ 5321 with adhering Tilia pollen Fig. S11 Bombus (Kronobombus) messegus Engel & Wappler, sp. nov., NHMMZ PE 1995/8792 with adhering Tilia pollen. Fig. S12 Bombus (Timebombus) palaeocrater Engel & Wappler, sp. nov., NHMMZ PE 1997/6137 with adhering Tilia pollen. Fig. S13 Extant bumble bees caught on silver lime (Tilia tomentosa) at the Botanical Garden of the University of Vienna, June 2024. Notes S1 The fossil record of Malvaceae flowers, morphological comparison of Tilia magnasepala sp. nov. and the ecology of extant Tilia. Notes S2 Systematic Palaeontology on Bombus Latreille. [file NPH-248-2111-s001.pdf]

New Phytologist **Supporting Information**

Article title: **24 million years of pollination interaction between European linden flowers and bumble bees**

Authors: Christian Geier, Michael S. Engel, Johannes M. Bouchal, Silvia Ulrich, Jürg Schönenberger, Dieter Uhl, Torsten Wappler, Sonja Wedmann, Loup Boudet, Friðgeir Grímsson

Article acceptance date: 07 August 2025

Supporting Figures and Notes

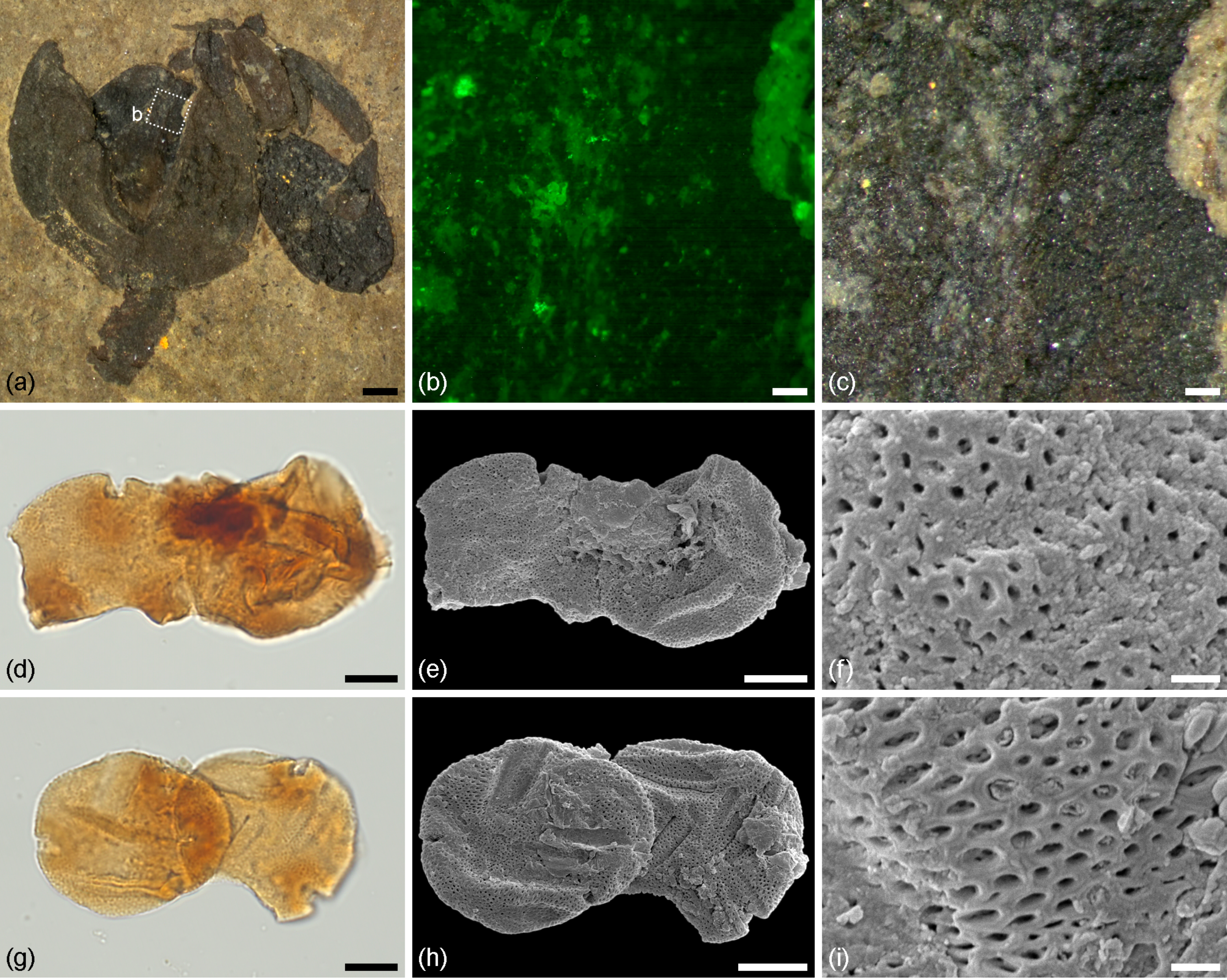

Fig.S1. Oligocene *Tilia magnasepala* C.Geier & Schönenb. flower bud from Enspel, Germany, NHMMZ PB 2013/ 5272. **(a)** overview of the flower bud; **(b)** center of the bud in **(b)**, in fluorescent light; **(c)** same area as in **(b)** but in white light; **(d, g)** light microscopy (LM) micrographs of in situ *Tilia* pollen extracted from the fossil *Tilia* bud; **(e, h)** scanning electron microscopy (SEM) overview micrographs of the same pollen grains as in **(d, g)**; **(f, i)** details of the exine surface in SEM. Scale bars = 1 mm (**a**), 0.1 mm (**b**, **c**), 10 µm (**d**, **e**, **g**, **h**), 1 µm (**f**,**i**).

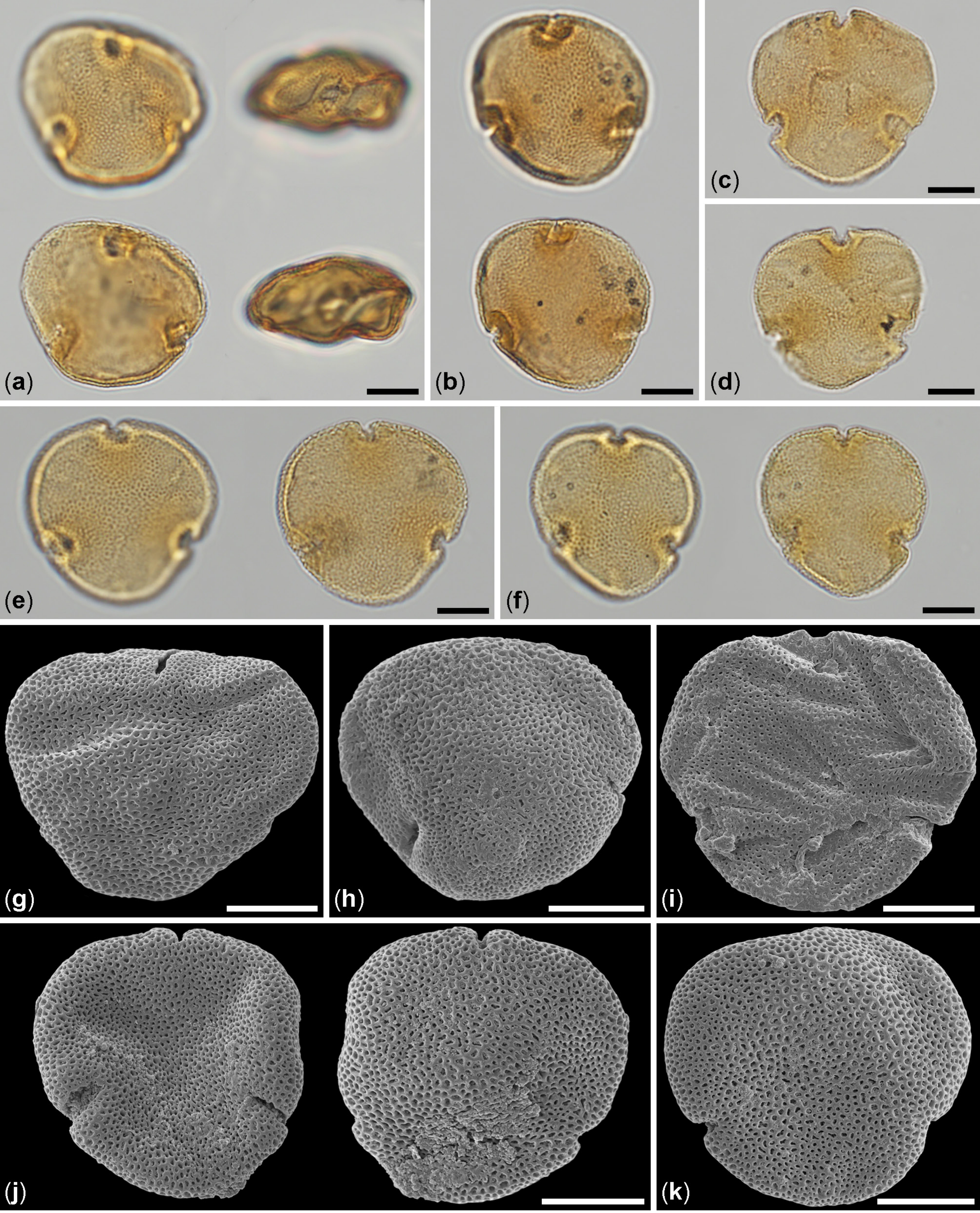

Fig. S2. Oligocene in-situ pollen extracted from flowers of *Tilia magnasepala* C.Geier & Schönenb. sp. nov. from Enspel, Germany, LM and SEM. Light microscopy micrographs (**a–f**) and scanning electron micrographs (**g–k**) of the same pollen grains, respectively. (**a, b, g, h**) NHMMZ PB 2014/5218-LS; (**c, d, i**) NHMMZ PB 2017/5055-LS; (**e, f, j, k**) NHMMZ PB 2017/5564-LS. **(a)** polar (left) and equatorial (right) views showing different focal planes; **(b)** polar view in different focal planes; (**c, d**) polar views; **(e, f)** polar views showing different focal planes; **(g–k)** polar views; **(j)** proximal (left) and distal (right) hemisphere of the same pollen grain. Scale bars = 10 µm.

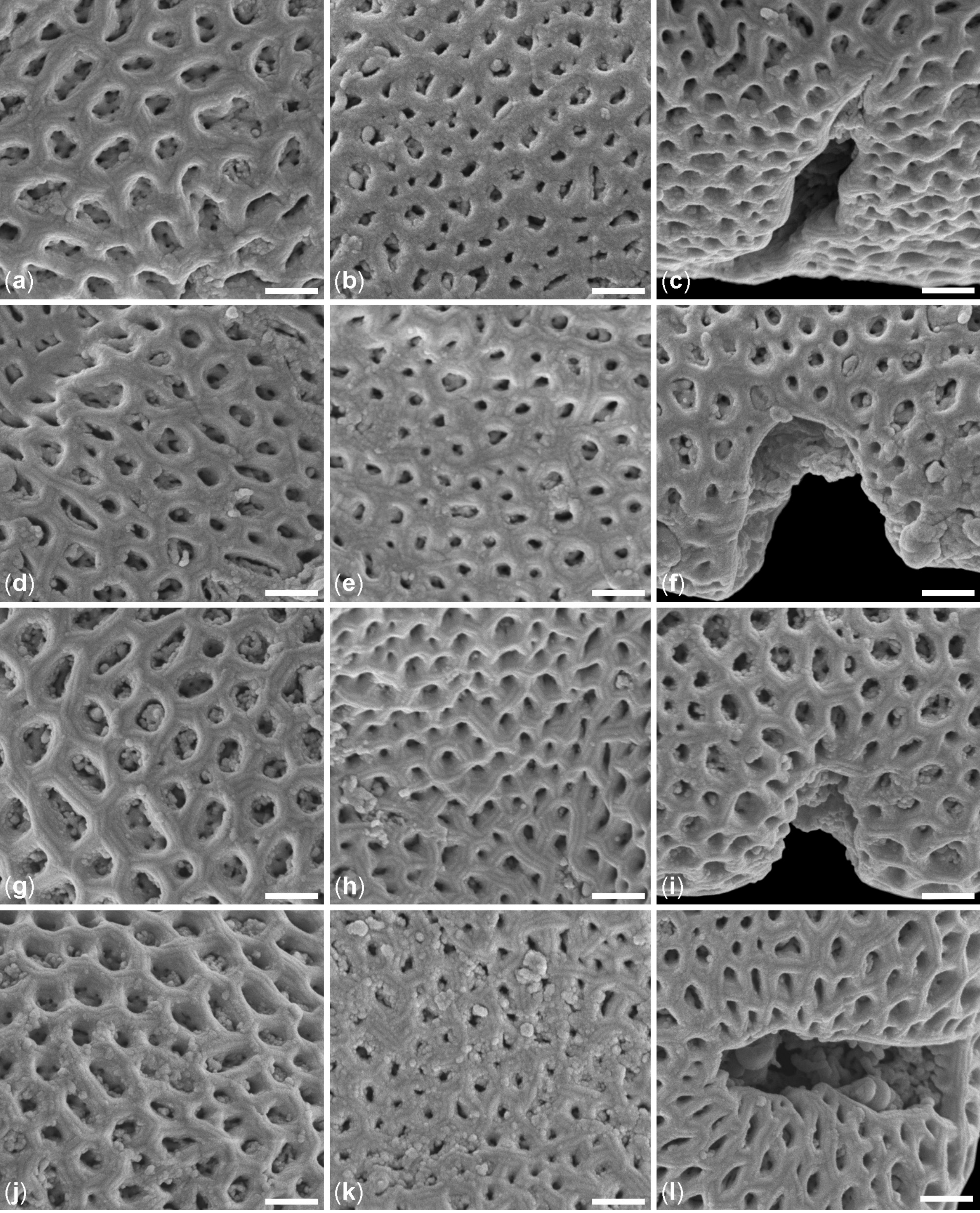

Fig. S3. Oligocene in-situ pollen extracted from flowers of *Tilia magnasepala* C.Geier & Schönenb. sp. nov. from Enspel, Germany, detail SEM micrographs. (**a–c)** NHMMZ PB 2014/5218-LS; (**d–f)** NHMMZ PB 2017/5055-LS; (**g–l)** NHMMZ PB 2017/5564-LS. (**a, b, d, e, g, h, j, k)** exine surface in polar area; (**c, f, i, l)** aperture. Scale bars = 1 μm.

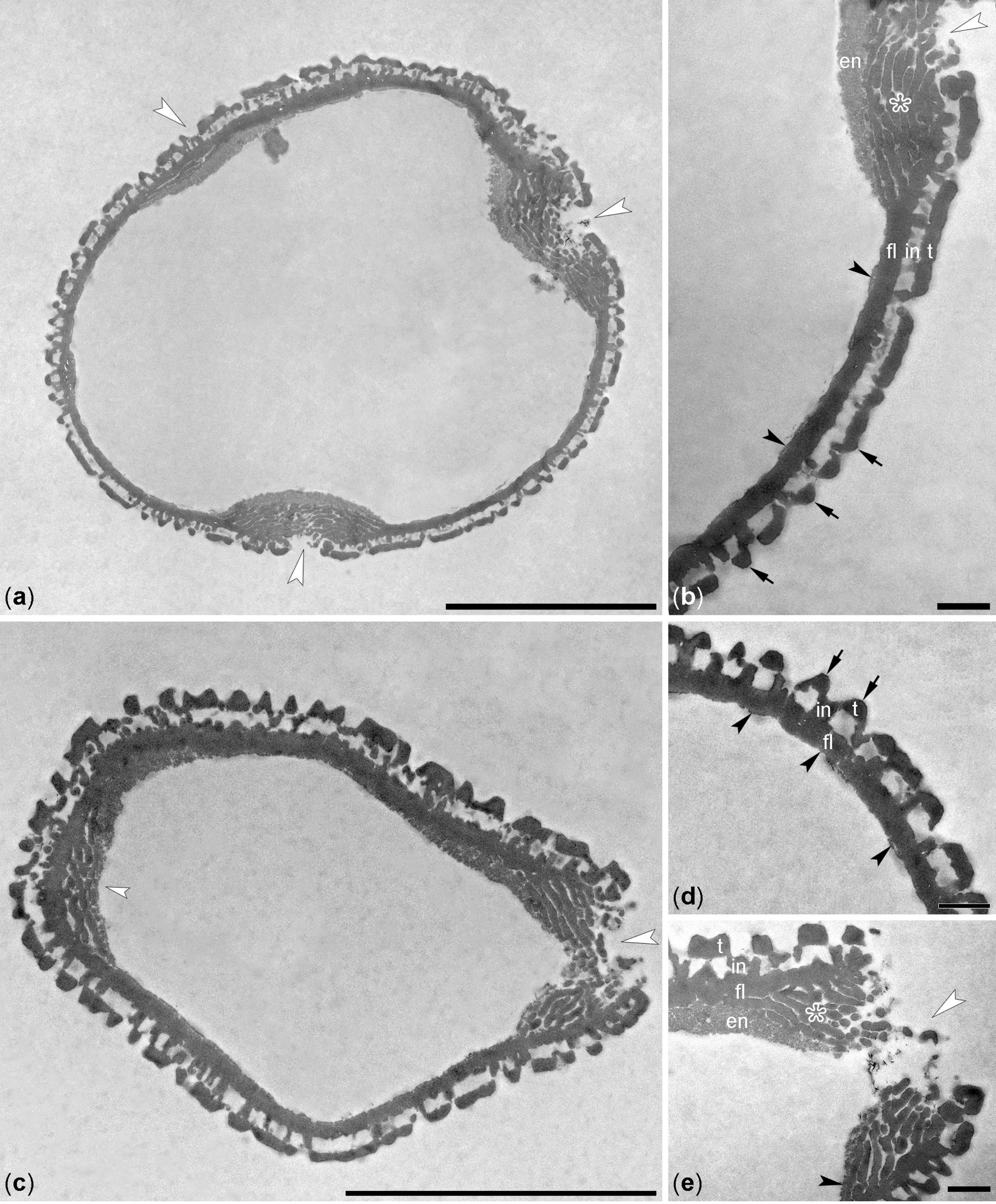

Fig. S4. TEM micrographs of in situ pollen grains extracted from flower of *Tilia magnasepala* C.Geier & Schönenb. sp. nov. NHMMZ PB 2017/5564-LS of Enspel, unstained. (**a**–**b**) Cross section of pollen grain in polar view; **(a)** Overview; **(b)** Detail showing transition from aperture to interapertural area; (**c**–**e**) Cross section of pollen grain in equatorial view. **(c)** Overview; **(d)** Detail of pollen wall; **(e)** Detail of aperture; Abbreviations: thin, continuous compact to spongy endexine (black arrowheads, en) thicker towards aperture area (white arrowheads), ektexine with semitectum (t) columellate-infratectum (i), thick, continuous-compact footlayer (fl) thicker and lamellated in aperture area (white asterisk), note striate supratectate ornamentation (black arrows); Scale bars = 10 µm (**a, c**), 1 µm (**b, d, e**).

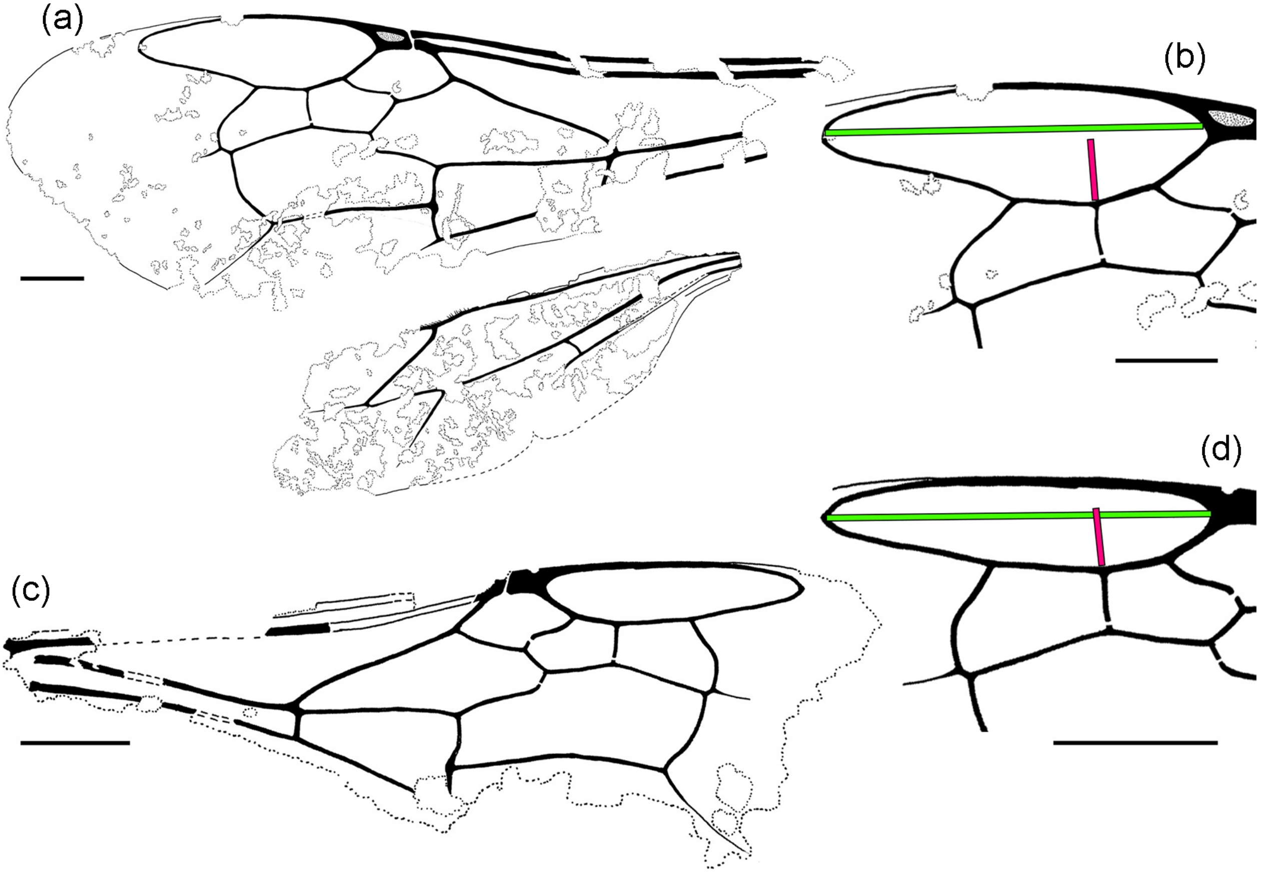

Fig. S5. Wing venation of Late Oligocene bumble bees (*Bombus* Latreille) from Enspel. **(a)** *Bombus* (*Kronobombus*) *messegus* subgen. et sp. nov.; **(b)** Detail of marginal cell of *B.* (*K.*) *messegus*; **(c)** *B.* (*Timebombus*) *palaeocrater* subgen. et sp. nov.; **(d)** Detail of marginal cell of *B.* (*T.*) *palaeocrater*. Green lines represent marginal cell tangent; red lines represent equivalent length of 1rs-m. Scale bars = 1 mm.

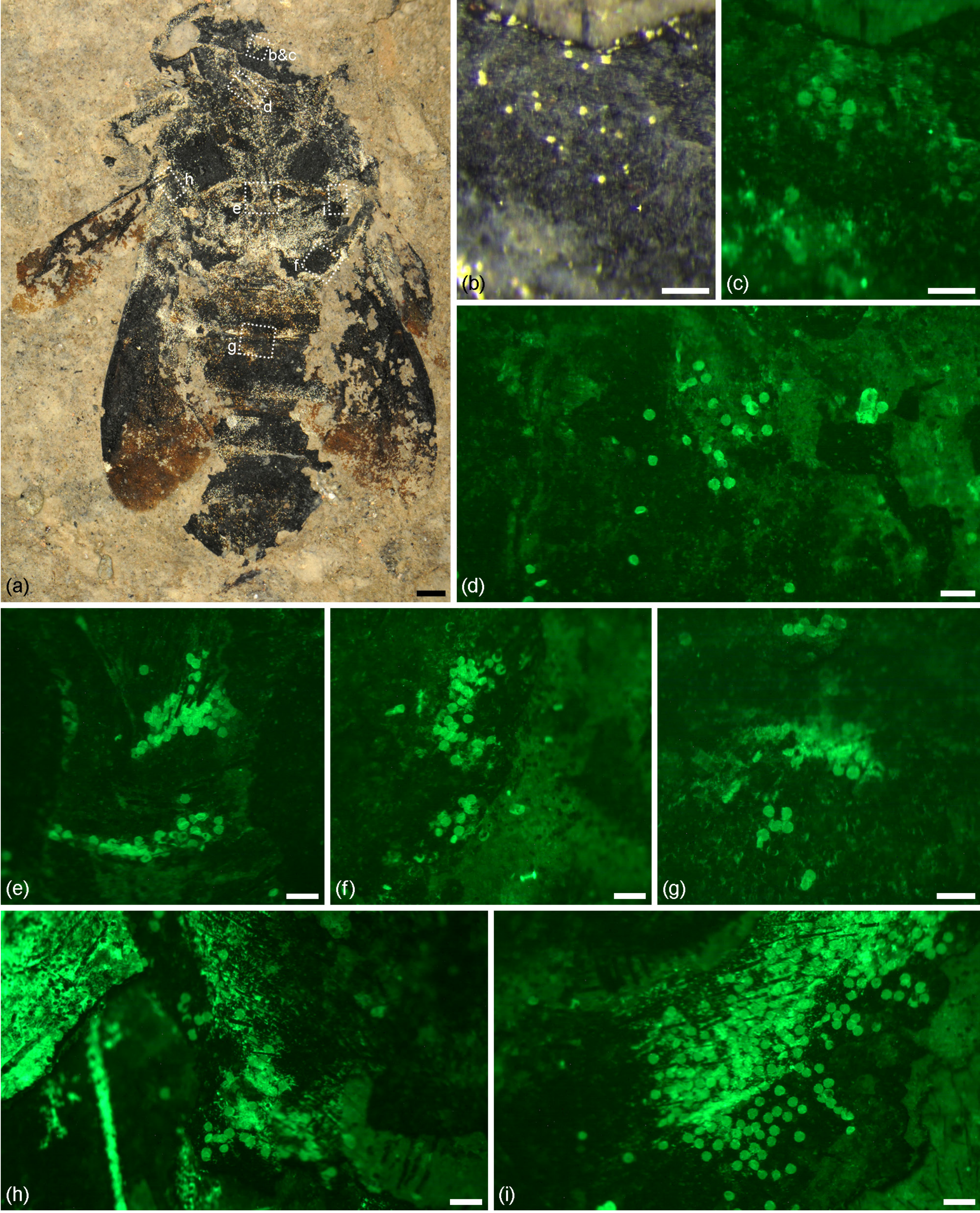

Fig. S 6. *Bombus* (*Kronobombus*) *messegus* Engel & Wappler, sp. nov., NHMMZ PE 2001/5215 with adhering *Tilia* pollen (**Fig. S 7a**–**o**). **(a)** overview of the bee; **(b)** head area, ventral; **(c)** same head area as in **(b)** but in fluorescent light; **(d)** thorax between the first and second pair of legs, ventral; **(e)** thorax between the second and third pair of legs, ventral; **(f)** thorax after the third pair of legs, ventral; **(g)** abdomen, centrally ventral; **(h)** attachment areas of the right wings, slightly ventral; **(i)** attachment area of the left wings, slightly ventral. Pollen fluoresces strongly in all images. Scale bars = 1 mm (**a**), 0.1 mm (**b**–**i**).

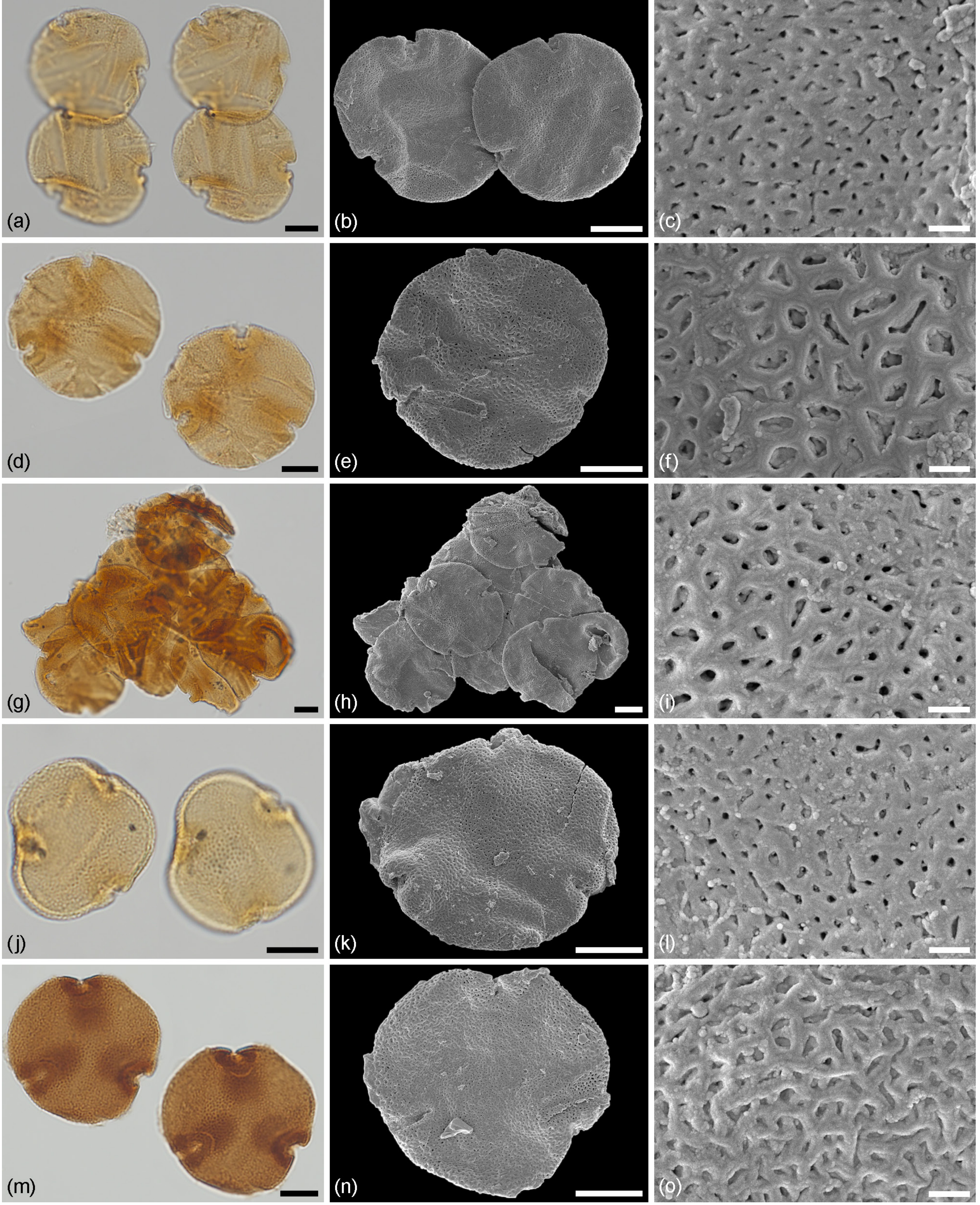

Fig. S7. Fossil *Tilia* pollen extracted from the exoskeleton of *Bombus* (*Kronobombus*) *messegus* Engel & Wappler, sp. nov. NHMMZ PE 2001/5215 (**Fig. S6**). **(a, d, g, j, m)** LM micrographs of fossil *Tilia* pollen and pollen clumps; (**b, e, h, k, n)** SEM micrographs of the pollen grain and pollen clumps in (**a, d, g, j, m**) respectively; (**c, f, i, l, o)** clos-up SEM micrographs of the exine surface of pollen grain and pollen clumps in (**b, e, h, k, n)** respectively. Scale bars = 10 µm (**a, b, d, e, g, h, j, k, m, n**), 1 µm (**c, f, i, l, o**).

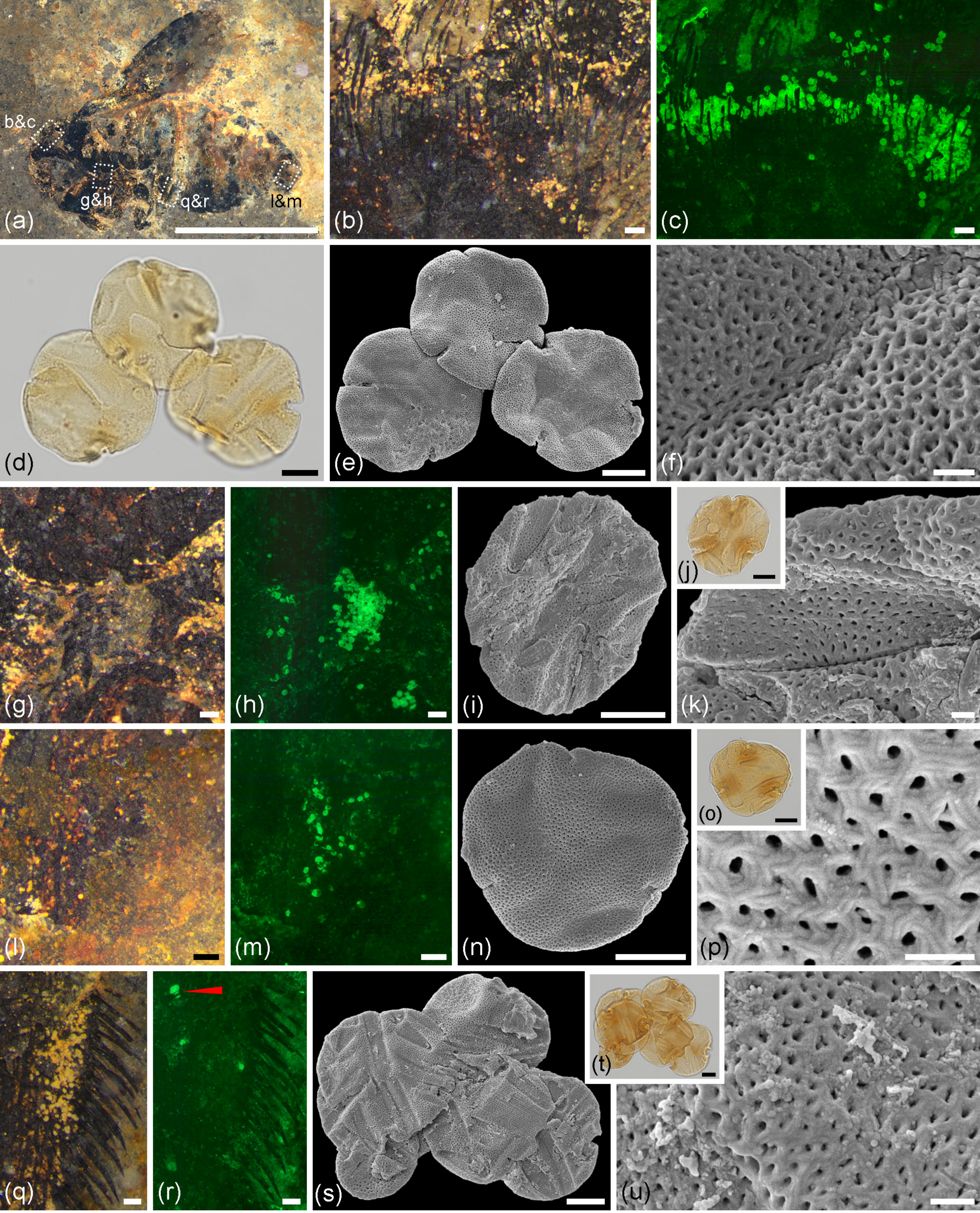

Fig. S8. *Bombus* (*Kronobombus*) *messegus* Engel & Wappler, sp. nov., NHMMZ PE 1995/5243 with adhering *Tilia* pollen (**d– f, i– k, n– p, s–u**). **(a)** overview of the bee; (**b**) head of the bee **(a)**; **(c)** same area as in **(b)** but in fluorescent light, pollen illuminates strongly; **(d–f)** *Tilia* pollen extracted from the head area in **(c)**; **(d)** *Tilia* pollen in LM; **(e)** same pollen as in **(d)** in scanning electron microscopy (SEM); **(f)** detail of the exine surface of pollen from **(e)**; **(g)** thorax area in between legs; **(h)** same area as in **(g)** but in fluorescent light; **(i–k)** *Tilia* pollen extracted from the thorax area in **(h)**; **(i)** *Tilia* pollen in SEM; **(j)** same pollen as in **(i)** in LM; **(k)** detail of the exine surface of pollen from **(i)**; **(l)** distal end if the abdomen; **(m)** same are as in **(l)**, fluorescent light; **(n–p)** *Tilia* pollen extracted from the abdomen in **(m)**; **(n)** *Tilia* pollen in SEM; **(o)** same pollen grain as in **(n)** in LM; (**k**) detail of the exine surface of pollen from **(n)**; **(q)** hindleg; **(r)** same hindleg in fluorescent light, pollen illuminates, red arrow point to pollen clump; **(s–u)** *Tilia* pollen extracted from the leg area in **(r)**; **(s)** *Tilia* pollen clump in SEM; **(o)** same pollen clump as in **(s)** in LM; **(u)** detail of the exine surface of pollen from **(s)**. Scale bars = 10 mm (**a**), 0.1 mm (**b, c, g, h, l, m, q, r**), 10 µm (**d, e, i, j, n, o, s, t**), 1 µm (**f, k, p, u**).

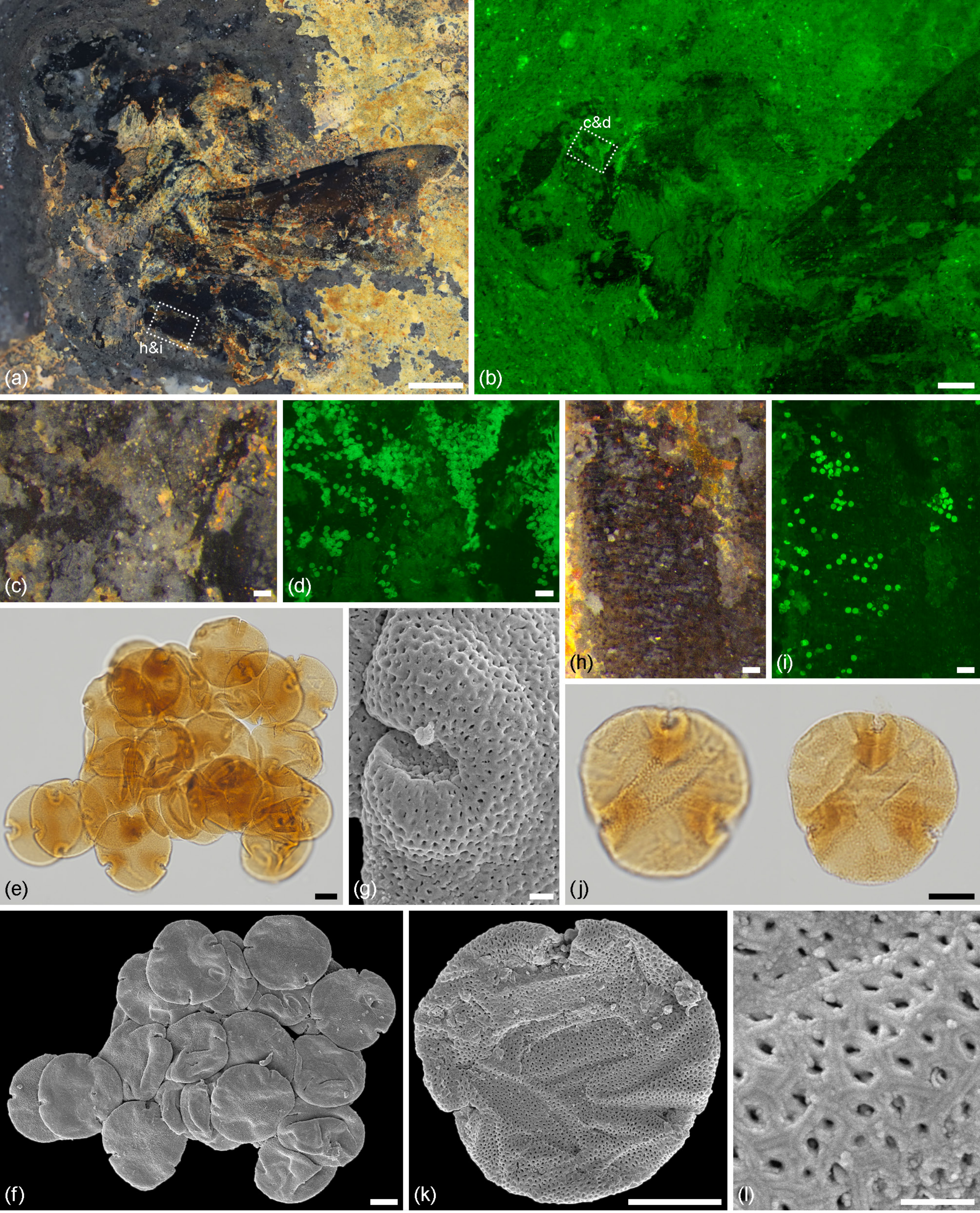

Fig. S9. *Bombus* (*Kronobombus*) *messegus* Engel & Wappler, sp. nov., NHMMZ PE 1995/5314 with adhering *Tilia* pollen (**e–g, j–l**). A full overview of the bee; (**b**) partial overview of the bee in fluorescing light; (**c**) head area; (**d**) same area as in (**c**) but in fluorescent light, pollen illuminates strongly; (**e–g**) *Tilia* pollen extracted from the head area in (**d**); (**e**) LM micrograph; (**f**) SEM micrograph of the pollen clump in (**e**); (**g**) detail of an aperture of pollen in (**f**); (**h**) ventral area of the abdomen; (**i**) same are as in (**h**), fluorescent light; (**j–l**) *Tilia* pollen extracted from the abdomen area in (**i**); (**j**) LM micrograph; (**k**) SEM micrograph of the same pollen grain as in (**j**); (**l**) detail of an aperture of pollen in (**k**). Scale bars = 2 mm (**a**), 1 mm (b), 0.1 mm (**c, d, h, i**), 10 µm (**e, f, h, k**), 1 µm (**g, l**).

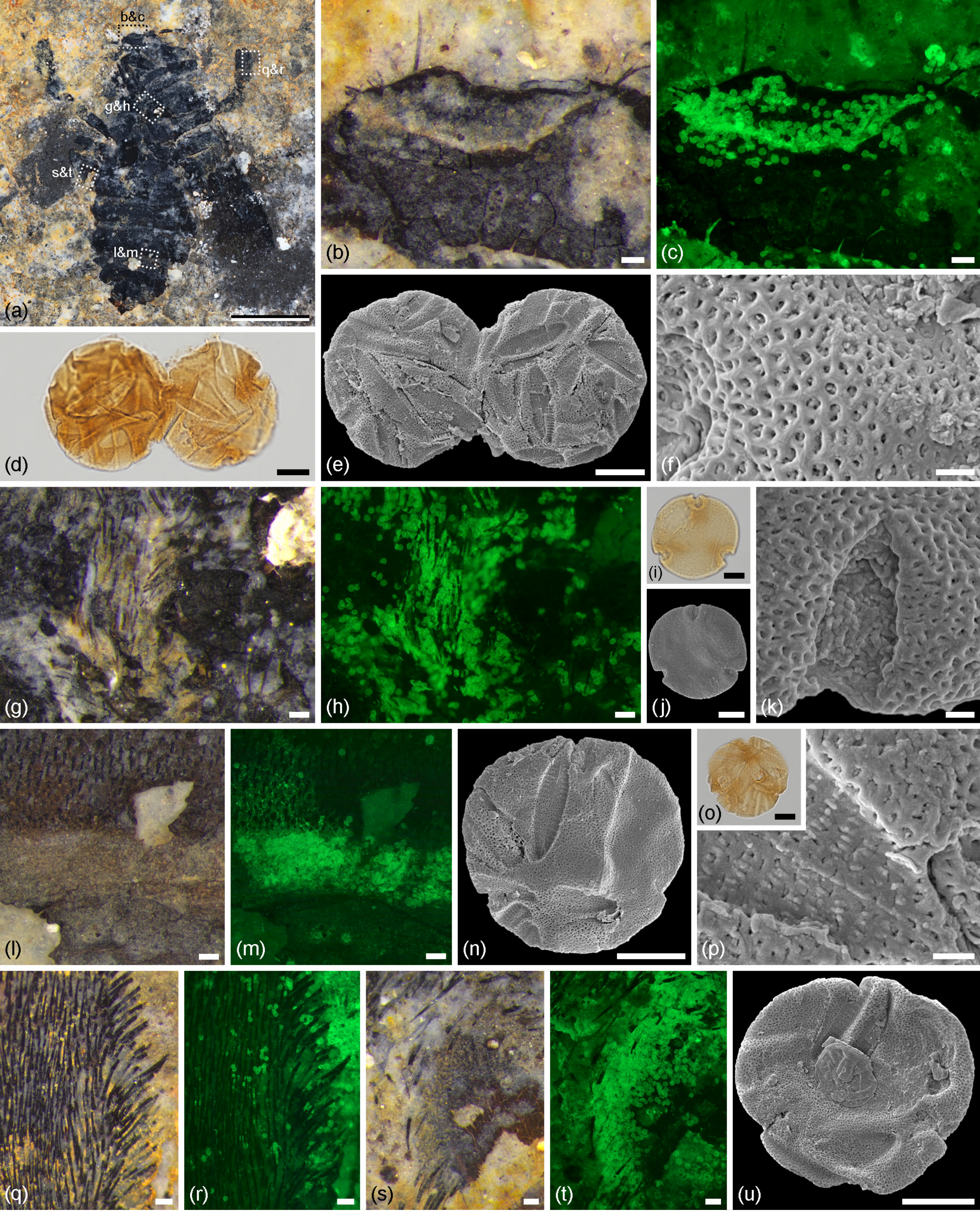

Fig. S10. *Bombus* (*Kronobombus*) *messegus* Engel & Wappler, sp. nov., NHMMZ PE 1995/ 5321 with adhering *Tilia* pollen (**e–g, i–k, m–p, u**). **(a)** overview of the bee; **(b)** head area; **(c)** same head area as in **(b)**, fluorescent light; **(c)** same area as in **(b)** but in fluorescent light, pollen illuminates strongly; **(d–f)** *Tilia* pollen extracted from the head area in **(c)**; **(d)** LM micrograph; **(e)** SEM micrograph of the pollen clump in **(d)**;**(f)** detail of exine surface of pollen in **(e)**; **(g)** thorax area in between legs; **(h)** same area as in **(d)** in fluorescence light; **(i–k)** pollen extracted from thorax in **(h)**; **(i)** LM micrograph; **(j)** SEM micrograph of the pollen grain in **(i)**; **(k)** detail of aperture of pollen in **(j)**; **(l)** abdomen;**(m)** same area as in **(l)** fluorescence light, pollen fluoresces brightly; **(n–p)** *Tilia* pollen extracted from the abdomen area in **(m)**; **(n)** SEM micrograph; **(o)** LM micrograph of the pollen in **(n)**; **(p)** detail of exine surface of pollen in **(n)**; **(q)** foreleg; **(r)** same foreleg as in **(q)** in fluorescence light, pollen grains in between setae; **(s)** hindleg; **(t)** same hindleg as in **(s)** in fluorescence light, large pollen clumps;**(u)** SEM micrograph of *Tilia* pollen extracted from the leg in **(t)**. Scale bars = 5 mm (**a**), 0.1 mm (**b, c, g, h, l, m, q–t**), 10 µm (**d, e, i, j, n, o, u**), 1 µm (**f, k, p**).

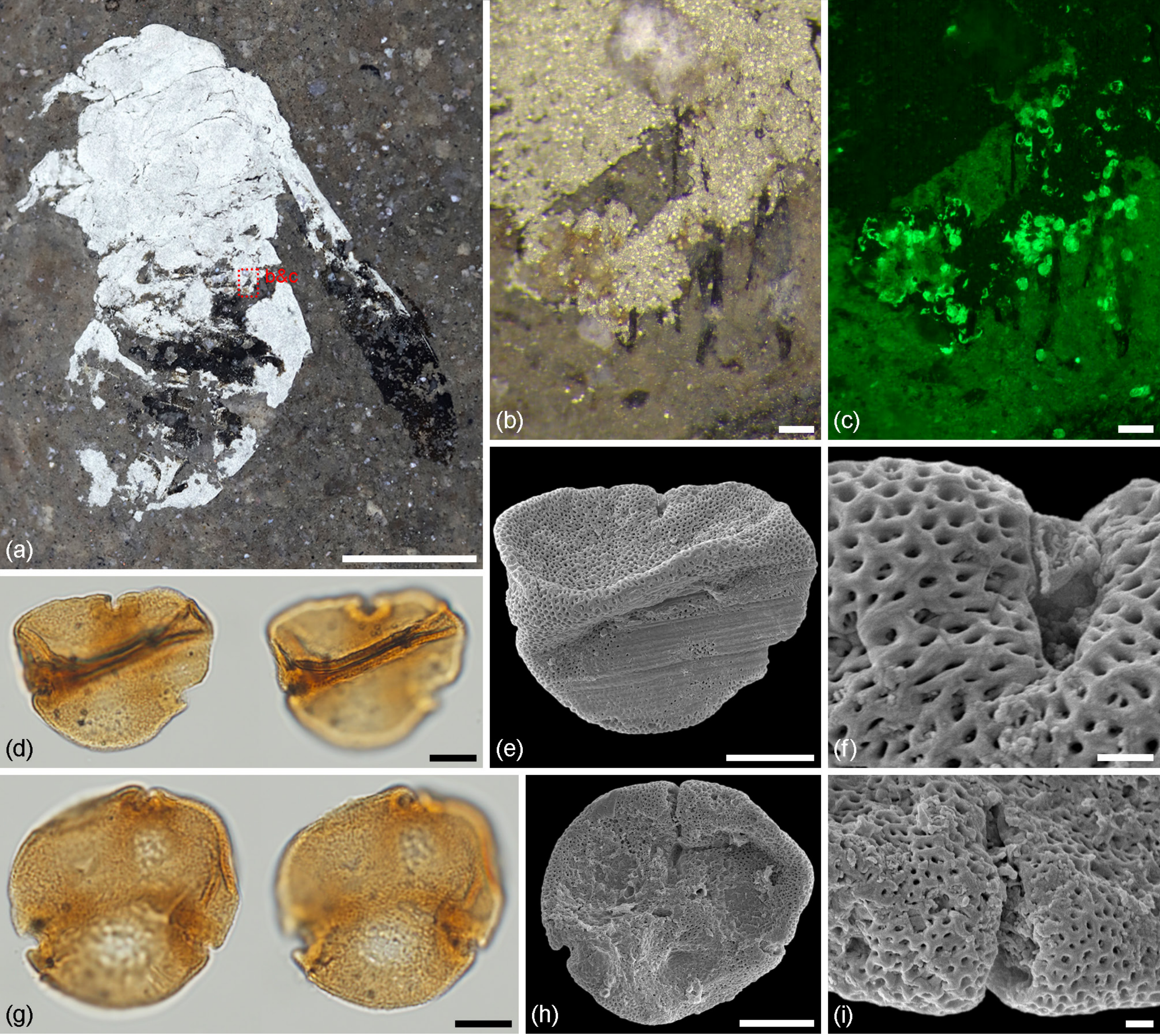

Fig. S11. *Bombus* (*Kronobombus*) *messegus* Engel & Wappler, sp. nov., NHMMZ PE 1995/8792 with adhering *Tilia* pollen (**d–i**). **(a)** overview of the bee; **(b)** abdomen area; **(c)** same abdomen area as in **(b)** but in fluorescent light, pollen is entangled in hairs and fluoresces strongly; **(d–i)** *Tilia* pollen extracted from the abdomen area in **(c)**; **(d, g)** LM micrographs of *Tilia* pollen in different focus sections; (**e, h**) SEM micrographs of pollen grains in **(d, g)**, respectively; **(f, i)** close up micrographs of the pollen grains in **(e, h)**. Scale bars = 5 mm (**a**), 0.1 mm (**b, c**), 10 µm (**d**, **e**, **g, h**), 1 µm (**f**, **i**).

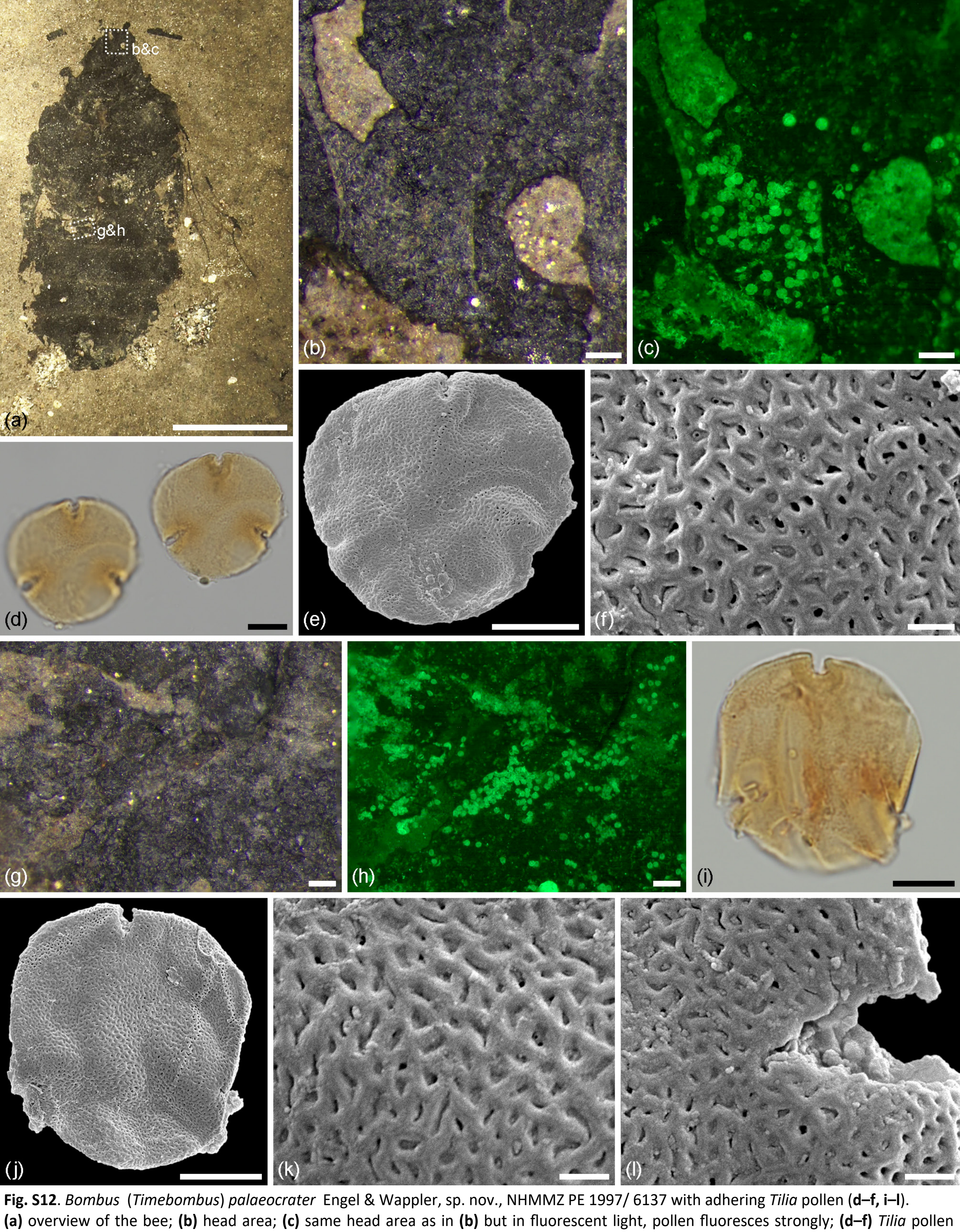

Fig. S12. *Bombus* (*Timebombus*) *palaeocrater* Engel & Wappler, sp. nov., NHMMZ PE 1997/ 6137 with adhering *Tilia* pollen (**d–f, i–l**).
(a) overview of the bee; **(b)** head area; **(c)** same head area as in **(b)** but in fluorescent light, pollen fluoresces strongly; **(d–f)** *Tilia* pollen extracted from the head area in **(c)**; **(d)** LM micrographs of *Tilia* pollen; **(e)** SEM micrograph of pollen grains in **(d)**; **(f)** close-up micrograph of the pollen exine in **(e)**; **(g)** abdomen; **(h)** same area of the abdomen as in **(g)**, fluorescent light, pollen fluoresces strongly; **(i–l)** *Tilia* pollen extracted from the abdomen area in **(h)**; **(i)** LM micrograph of a *Tilia* pollen grain; **(j)** SEM micrograph of the same pollen grain as in **(i)**; **(k)** close-up of the exine surface; **(l)** close-up of the aperture. Scale bars = 5 mm (**a**), 0.1 mm (**b, c**), 10 µm (**d, e, i, j**), 1 µm (**f, k, l**).

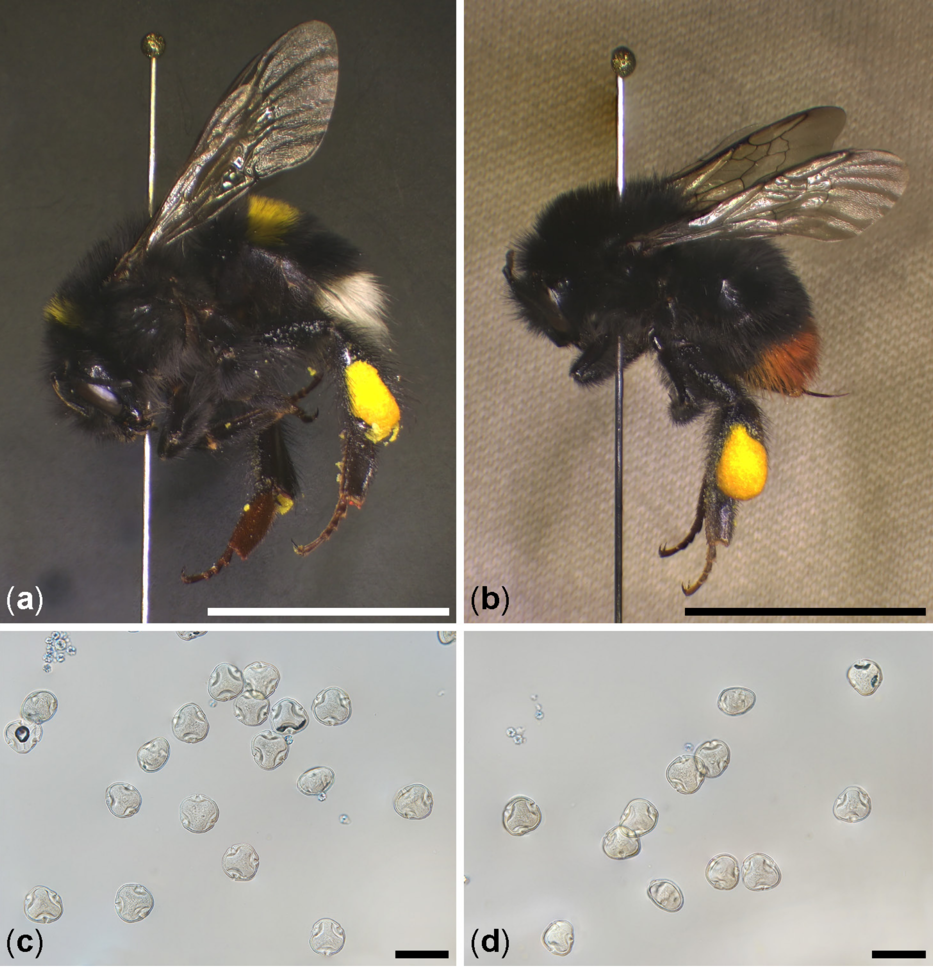

Fig. S13. Extant bumble bees caught on silver lime (*Tilia tomentosa*) at the Botanical Garden of the University of Vienna, June 2024. (**a**) *Bombus* subgen. *Bombus*. sp. (**b**) *Bombus* subgen. *Melanobombus*. sp. (**c**) *Tilia* pollen extracted from the ambrosia on *Bombus* subgen. *Bombus*. sp. (**d**) *Tilia* pollen extracted from the ambrosia on *Bombus* subgen. *Melanobombus*. sp. Note the big ambrosia clumps (>99% pollen of *Tilia*) on the hindlegs of both specimens. Scale bars = 1 cm (**a, b**), 50 µm (**c, d**).

Note S1

The fossil record of Malvaceae flowers, morphological comparison of *Tilia magnasepala* sp. nov. and the ecology of extant *Tilia*

The fossil record of flowers/buds of Malvaceae

We are currently aware of only a few published records on flowers/flower buds of Malvaceae. These include specimens assigned to the subfamilies Bombacoideae, Sterculioideae, and Tilioideae (Supporting Information, Table S4). The records of Bombacoideae comprise *Eriotheca prima* Duarte, a flower from the middle to late Eocene of Brazil (Duarte, 1974; Mello *et al.*, 2000), *Bombax sepultiflorum* Saporta, flowers from the Oligocene of France (De Saporta, 1862, 1877), and *B. asiatica* Hazra, Bera et Khan, a flower from the Pliocene of eastern India (Hazra *et al.*, 2023). Records of Sterculioideae comprise flowers of *Florissantia ashwilli* Manchester, *F. quilchenensis* (Mathewes & Brooke) Manchester, and *F. speirii* (Lesquereux) Manchester, some with in-situ pollen, from various middle Eocene to Oligocene localities in North America, mostly of the USA but also Canada (Manchester, 1992). The records of Tilioideae comprise the genera *Craigia* and *Tilia* as well as flowers assigned only to the subfamily Tilioideae from the Eocene of Messel, Germany (Geier *et al.* 2025). Numerous flowers/buds, some with in situ/adhering pollen, of *Craigia bronnii* (Unger) Z. Kvaček, Bůžek et Manchester are reported from various Miocene localities in both Germany and Czechia (Mai, 1961, 2000; Mai & Walther, 1991; Pingen & Gregor, 1994; Pingen *et al.*, 2001; Kvaček *et al.*, 2002; Zetter *et al.*, 2002; Kvacek, 2004). *Tilia* is also represented by a flower and potentially a floral bud from the Late Eocene/Early Oligocene of North America (Hall & Swain, 1971; Manchester, 1994) and two bundles of stamens with in-situ pollen from the Oligocene of England (Chandler, 1957). None of these flowers or in-situ/adhering pollen correspond to the fossil flowers of *Tilia* and in-situ/adhering pollen described herein, but see later sections on comparison to relevant fossil and extant flowers and pollen. For Enspel so far, no flowers assignable to *Tilia* have been reported (Uhl, 2015).

The in-situ/adhered pollen of *Tilia* compared to modern equivalents

The pollen morphology of *Tilia* has been investigated thoroughly by various authors using all traditionally applied microscopy techniques (LM, SEM, and TEM; *e.g.*, ((IBSCIB-CAS), 1982; Beug, 2004; Perveen *et al.*, 2004; Auer *et al.*, 2024; Geier *et al.*, 2025). The pollen morphology is, similar to the floral morphology, relatively uniform across the genus (Supporting Information 01, Table S2). Pollen of *Tilia* is medium sized, has a polar axis of 17–36 µm in LM, 16–26 µm in SEM, and an equatorial diameter of 29–52 µm in LM and 28–42 µm in SEM. All species have oblate, convex-triangular/circular pollen, with three brevicolporate apertures, a more or less obtuse to right angled, lense to crescent-shaped thickening at the apertures made of nexine (costae) and a reticulate ornamentation in LM, and microreticulate to nanoreticulate to perforate ornamentation in SEM. Some species (*T. americana*, *T. endochrysea*, *T. miqueliana*, and *T. tomentosa*) additionally have a striate suprasculpture on the muri. Exine thickness ranges from 1.6–2.5 µm, with the sexine being equally or slightly thicker than the nexine. The pollen wall is differentiated into a semitectate tectum, a columellate infratectum, a thick compact-continuous footlayer, and a thinner endexine. Some species have slightly larger or smaller pollen grains but that could also be caused by preparation methods (*e.g.*, LM: acetolysed and in glycerine vs in silicon oil; SEM: acetolysed

vs. (critical-point-) dried). The fossil pollen of *T. magnasepala* falls completely within the range of extant pollen for *Tilia* (Supporting Information, Table S2). The P/E ratio, outline and shape, aperture condition, number of apertures, and the thickening of nexine around the apertures (costae) of the fossil pollen is consistent with pollen of extant *Tilia* (Table S2). Small differences can be observed in the exine thickness, the fossil pollen being on the thinner end of the spectrum and recent pollen from *Tilia* generally a bit more substantial. This could have a taphonomic reason, since the fossils are compressed and underwent thermal alteration from the basalt that has been deposited directly on top of the fossiliferous lake sediments (Felder *et al.*, 1998). Regarding the ornamentation, all extant taxa have pollen with a reticulate exine sculpture, some with larger lumina and others smaller. The distal and proximal hemisphere of pollen grains of *Tilia* are known to be slightly different, the proximal side with a finer reticulum than the distal side (Beug, 2004). The same morphological characteristics can be observed in the fossil pollen (Fig. 3j). The most striking feature of the exine ornamentation is the striate suprasculpture on the muri of the reticulum of pollen extracted from *T. magnasepala*. Such suprasculpture can only be observed with SEM and has so far only been documented for pollen of three extant species of *Tilia*: *T. americana*, *T. endochrysea*, and *T. tomentosa*, all from different sections (Anastraea, Endochrysea, and Astrophilyra, respectively [sensu (Pigott, 2012)]). Still, we are unable to affiliate the fossil flowers to a certain section within *Tilia*.

***Tilia* in the Late Oligocene of Enspel**

Flowers are fragile structures and following taphonomic research on extant and fossil plant assemblages, summarized by (Spicer, 1989, 1991) and (Ferguson, 1993), their preservation in the Enspel sediments suggest a short transport prior to rapid sedimentation. This indicates that trees of *Tilia* were growing close to the margin of the ancient Enspel lake or at small streams running into it. Therefore, during the Oligocene, *Tilia* was probably part of the lowland forests bordering the Enspel lake. Today, *Tilia* has a wide ecological range regarding habitat (incl. substrate, temperature, precipitation) depending on the individual species latitudinal / longitudinal distribution as well as elevation occurrence (Pigott, 2012). In North America, *Tilia* is a forest component and occurring along lake shores and streams, from lowlands to about 800 m above sea level (Hanes, 2015). In Europe and Western Asia, *Tilia* are among the most widely distributed trees of the temperate lowland forests, but can be found from sea level up to an elevation of about 1500 m (the Alps) depending on their geographic occurrence (Pigott, 2012). In East Asia, trees of *Tilia* are not prominent in lowland vegetation units, but occur in evergreen or mixed evergreen and deciduous forests at elevation between 600 and 4000 m (Ya *et al.*, 2007). Considering the above, the trees of *Tilia* thriving around the Late Oligocene Enspel lake fit better within the eco-range of both North American and European/Western Asian species of *Tilia*, rather than those of East Asia.

There are currently two major works on the paleoflora from Enspel. Herrmann *et al.* (2009, 2010), provided a detailed description and photographic documentation of both spores and pollen from this locality, and Köhler & Uhl (2014) delivered a similar work on the macroflora (leaves, fruits, seeds). The combined taxon list from these and additional publications suggest a fairly rich paleoflora composed of various mosses, lycopods, and ferns, as well as a number of gymnosperms and numerous different angiosperms (Table S4 to S6). The genera encountered, especially those of woody angiosperms (trees, shrubs, lianas, etc.), are mostly suggestive of plants occurring in today's Temperate to Subtropical Forest regions of Eastern North America and East Asia (Table S7, S8; ('eFloras', 2008; POWO, 2024). Therefore, we visualise the paleovegetation during the Late Oligocene of Enspel as forest dominated, composed of mixed conifer and broadleaved trees. The broadleaved elements were

characterised by diverse deciduous canopy trees (*e.g., Acer*, *Alnus*, *Betula*, *Carya*, *Carpinus*, *Cercidiphyllum*, *Cornus*, *Craigia*, *Fagus*, *Fraxinus*, *Juglans*, *Liquidambar*, *Nyssa*, *Ostrya*, *Platanus*, *Pterocarya*, *Quercus*, *Tilia*, *Ulmus*, and *Zelkova*) and smaller understorey trees and shrubs (*e.g.*, *Crataegus*, *Diospyros*, *Salix*), as well as deciduous or evergreen lianas and climbers (*e.g.*, *Ampelopsis*, *Actinidia*, *Hedera*, *Parthenocissus*, *Vitis*). Evergreen small trees and shrubs (*e.g.*, *Ilex*, *Itea*, *Mahonia*, *Meliosma*, *Myrica*, *Pyracantha*, *Rosa*, *Sloanea*, *Symplocos*) were also part of the understorey. Furthermore, based on the ecological characteristics of potential modern analogues of the fossil plants from Enspel, we assign the fossil floral elements to eight different paleovegetation units (but see (Köhler & Uhl, 2014)) occurring within and around the ancient Enspel lake (Supporting Information, Table S7, S8). This categorisation suggests that the paleoflora of Enspel is mostly composed of remains from plants that thrived in the vegetation units: 1) Backswamp and riparian forest and temporally flooded lake margins, 2) Well-drained lowland forest and lake margin, and 3) Well-drained lowland and foothill forest (Table S8). The latter two units are where we assume that trees of *Tilia* were part of the forest vegetation.

The climatic and biome preferences of extant *Tilia* support that assessment for the vegetation surrounding the former crater lake of Enspel (Geier *et al.*, 2025). The genus grows in warm to cold temperate climates (*Cfa, Cfb, Csa, Cwa, D*-climates), as part of a mixed broadleaf and conifer lowland forest (Olson *et al.,* 2001). Likewise, to its modern counterparts, *T. magnasepala* grew in the lowlands and slopes relatively close to the lake, where flowers were visited by bumble bees, some flowers dropped or were torn from the branches and fell into the lake or were transported over a short distance by surface run-off or by rivulets.

Note S2

Systematic Palaeontology on *Bombus* Latreille

In the following accounts the morphological terminology follows that of Engel (2001a) and Michener (2007).

Tribe Bombini Latreille, 1802
Genus *Bombus* Latreille, 1802, *s.l.*

Bombus comprises 294 extant species colloquially known as bumble or humble bees and represents the only genus of the corbiculate tribe Bombini (Michener, 2007; Engel & Rasmussen, 2021). The fossil record of bumble bees is poor (Barden & Engel, 2021), with comparatively few species and most of those known are imperfectly understood as some species are known only from wings or existing descriptions are rather meagre in meaningful data (Michez *et al.*, 2012; Wappler *et al.*, 2012; Dehon *et al.*, 2014, 2019; Prokop *et al.*, 2017). Regardless, extinct species attributed to Bombini range in age from latest Eocene to late Miocene and include some previously ascribed to other genera (Dehon *et al.*, 2019). At least one of these species is misplaced. *Oligobombus cuspidatus* Antropov from the Priabonian (Eocene) of the Isle of Wight is certainly not a bumble bee and although it is quite incompletely characterized owing to the nature of its preservation, what is preserved demonstrates that there is nothing supporting an attribution to Bombini. Naturally, character data from the hind wing and body would be critical for a more confident assignment but at least the combination of a lack of a distinct bow in the anterior half of 2Rs, the broad marginal cell, and the more posterior origin of 3Cu (*i.e.*, 2cu-a comparatively short relative to 2Cu) are more indicative of the Electrobombini, which

is also known only from the Eocene, and are never found in combination in Bombini. Accordingly, we transfer *Oligobombus* Antropov to the Electrobombini, a tribe which is either an extinct sister group to Bombini or a stem group to all other corbiculates excluding Euglossini and Bombini (Engel, 2001a,b; Schultz *et al.*, 2001; Engel & Rasmussen, 2021). Indeed, one possible set of relationships could be {[(Bombini + Electrobombini) *Protobombus*] (Melikertini + Meliponini)} {*Electrapis* (*Thaumastobombus* + Apini)}, although it is also possible that the fossils continue to corroborate the traditional concept of relationships for Corbiculata (Engel & Rasmussen, 2021). Numerous fossil corbiculates await formal description (Engel unpubl. data) and once these are covered a revised phylogenetic study of living and fossil corbiculate bees will be warranted.

The removal of *Oligobombus* to Electrobombini leaves *Calyptapis florissantensis* Cockerell from the Eocene-Oligocene boundary of the Florissant Formation (USA) as the earliest evidence of bumble bees (Dehon *et al.*, 2019). Here we consider *Calyptapis* as a subgenus of *Bombus* as there is a rather continuous transformation series of character-states represented by *Calyptapis*, the new species from Enspel (Fig. 5), and the Early Miocene species of Bes Konak, Turkey (Fig. 5) (Dehon *et al.*, 2019). The species from Enspel described here represent two new subgenera, *Kronobombus* subgen. nov. and *Timebombus* subgen. nov., the latter with a noticeably more derived venation. An alternative system could be proposed recognizing *Calyptapis* Cockerell and *Kronobombus* as genera with *Bombus* restricted to those groups with a narrow marginal cell compared to the submarginal cells (*i.e.*, marginal tangent not crossed by length of 1rs-m: *vide infra*). We have opted for a broader circumscription of *Bombus* encompassing these stem groups as at least *Kronobombus* has a hallmark apomorphy of bumble bees, *i.e.*, the complete loss of the hind wing jugal lobe, even if other aspects of the wing venation are symplesiomorphic (*e.g.*, broad marginal cell relative to submarginal cells, form of third submarginal cell, greatly elongate hind wing M+Cu). The species of *Calyptapis* is the earliest to exhibit the characteristic bombine bowed 2Rs (hence our choice to include it within *Bombus*), while retaining an obliquely sinuate 2rs-m with posterior extension of third submarginal cell, second medial cell with anterior margin noticeably 'peaked' (*i.e.*, 3M and 4M noticeably angled relative to each other), 2m-cu long and gently and weakly arched (rather than short and arching proximally in posterior portion), and marginal cell broad compared to submarginal cells (*i.e.*, tangent from medioapical point of pterostigma to apex of marginal cell demarcating space at most as wide as length of 1rs-m at same transverse tanget of this crossvein: rather than in crown-group *Bombus* and more derived extinct subgenera where the length of 1rs-m crosses the marginal cell tangent). From Enspel there are two species, one of which intermingles plesiomorphies of *Calyptapis* with a seemingly more derived third submarginal cell whereby 2rs-m is not sinuate and instead straight anteriorly and simply arched posteriorly and 3Cu originates in a medial position from 2Cu/2cu-a. It also combines in the hind wing a plesiomorphically elongate 2M+Cu with the apomorphic loss of the distal abscissa of M (*i.e.*, the "indica vein" of literature on Apini), a trait typical of Bombini. The second species has more derived features in that the marginal cell is narrowed and 2rs-m is less oblique and more weakly curved thereby shortening the posterior border of the third submarginal cell relative to the anterior border.

Kronobombus Engel, subgen. nov.

Type species: *Bombus* (*Kronobombus*) *messegus* Engel et Wappler, sp. nov.

Diagnosis: This subgenus is seemingly an early-diverging lineage of *Bombus* but perhaps not as much as *Calyptapis*, although both subgenera have the characteristic bombine bowed 2Rs (Figs 5, S5a, b). Like *Calyptapis*, the depth of the marginal cell relative to the submarginal cells is broad, such that the marginal tangent (as described above) is not crossed by the distance of 1rs-m within the posterior of the cell (Fig. S5b) (in *Timebombus*, *Paraelectrobombus* Nel & Petrulevičius, and extant subgenera the tangent is crossed by the length of 1rs-m; the marginal cells of Electrobombini, inclusive of *Oligobombus*, are like those of *Calyptapis* and *Kronobombus*: Fig. 5). It also retains a long and weakly curved 2m-cu and 'peaked' anterior border to the second medial cell (anterior border either

continuous across 3M and 4M or weakly angled in *Timebombus*, *Paraelectrobombus*, and is typical for extant subgenera) (Fig. S5a). Like *Calyptapis* 2rs-m is strongly oblique (Fig. 5), with the posterior portion of the third submarginal cell extended apically at the posterior arch but unlike *Calyptapis* 2rs-m is not sinuate and instead straight in its anterior half (Fig. 5). In addition, the prestigma is as long as the pterostigmal width and 2M is notably angled posteriorly relative to Rs+M in *Kronobombus*, while in *Calyptapis* the prestigma is distinctly shorter than pterostigmal width and 2M is only weakly angled posteriorly relative to Rs+M (Fig. 5). Quite interestingly, the hind wing of *Kronobombus* lacks a jugal lobe as well as a distal abscissa to M (= indica vein of Apini), distinctive character states of *Bombus*, while 2M+Cu is notably elongate, being about 1.75× the length of 1M (Figs 5, S5a) (in this regard similar, putatively symplesiomorpically, to the hind wing of *Electrobombus samlandensis*: *vide* Engel, 2001a). As is typical for *Bombus*, the mandible retains outer mandibular grooves, is broadly curved apically, and the labrum is transverse.
Etymology: The new subgeneric name is a combination of Ancient Greek ***χρόνος*** (*khrónos*, meaning, "time") and *Bombus* (itself taken from ***βόμβος/****bómbos*, meaning, "buzzing"). The gender of the name is masculine.
ZooBank LSID: urn:lsid:zoobank.org:act:F16890FE-E017-4075-8968-CD7AFB2BBFF4

Bombus (*Kronobombus*) *messegus* Engel & Wappler, sp. nov. (Figs 5, 6a-f,; Supporting Information, Figs S5a, b, S6–S11)
Holotype (*hic designatus*): NHMMZ PE 2001/5215a-LS (Fig. 6a) and NHMMZ PE 2001/5215b-LS (Fig. 6b) (part and counterpart).
Paratypes: NHMMZ PE 1995/5321-LS (Fig. 6c), NHMMZ PE 1995/5243a-LS (Fig. 6d) and NHMMZ PE 1995/5243b-LS (part and counterpart), NHMMZ PE 1995/5314-LS (Fig. 6e).
Additional material: One specimen is attributed to this species but is excluded from the type series owing to its general state of preservation, NHMMZ PE 1995/8792-LS (Fig. 6f).
Type locality: Enspel, Rhineland-Palatinate, Germany.
Type stratum: Enspel Fm, Layer S14; digging site G19B.
Age: 24.56±0.04 Ma, Chattian, Oligocene.
Etymology: The specific epithet is the Ancient Greek adverb ***μεσσηγύς*** (*messēgús*, meaning, "in the middle").
ZooBank LSID: urn:lsid:zoobank.org:act:D8022166-6FAB-4CB9-92C8-2AFFEFE8D129
Diagnosis: As for the subgenus (*vide supra*).
Description: ♀: Total body length (as preserved) 22.4 mm (paratype – PE 1995/5243: 20.7 mm, PE 1995/5314: 13.2 mm, PE 1995/5321: 18.9 mm), forewing length (as preserved) ca. 13.6 mm (paratype PE 1995/5243: ca. 14 mm, PE 1995/5314: ca. 12 mm, PE 1995/5321: ca. 9.5 mm). Integumental sculpture and coloration not preserved [integument represented by taphonomically altered carbonaceous compression; bee's integument likely black in life]; body robust with abundant shaggy setae [coloration as preserved dark brown to black, whether colour patterns were present in life cannot be determined]. Head broader than long [only observable obliquely in paratype 5234a]; labrum transverse [surface details not discernible]; malar space apparently shorter than wide [based on paratype 5234a]; mandible with apical margin curved (not oblique) [paratype 5321, apex of mandible in holotype missing], with outer mandibular grooves present [holotype and paratype 5321].
Mesosoma robust, much broader than head, maximum width 9 mm as preserved (paratype PE 1995/5243: ca. 8 mm, PE 1995/5321: ca. 6 mm), fringe of setae particularly elongate on mesoscutellum [observable in paratype 5314]. Legs with abundant setae; metatibia comparatively narrow, about as long as metatrochanter + metafemur, with fringes of setae bordering corbicular surface, with profundal setae (seemingly over proximal third) [most easily visible in paratype 5234a]; metabasitarsus longer than wide, length approximately 1.5× apical width, about as broad as metatibia apically, margins not converging apically, apical margin straight, apical angle rounded, margins with fringes of short

bristles, without fringe of elongate setae (such fringe present in *Mendacibombus* Skorikov) [most easily visible in paratype 5234a].

Forewing with membrane darkly infumate, lighter apically beyond closed cells; 1M slightly proximal to 1cu-a, straight; 1Rs short, shorter than Rs+M; Rs+M slightly longer than 2M; 2Rs bowed anteriorly; pterostigma slightly longer than wide, prestigma as long as pterostigmal width; marginal cell broad, apically slightly offset from anterior wing margin, apex narrowly rounded, not appendiculate, free portion of marginal cell shorter than portion bordering submarginal cells; r-rs slightly longer than 3Rs; 4Rs longer than 3Rs; 1m-cu entering second submarginal cell slightly proximal midlength, thus 2M slightly shorter than 3M; 3M angled at juncture with 4M (*i.e.*, second medial cell 'peaked'), shorter than 4M; 1rs-m straight; 2rs-m oblique, straight anteriorly, gently arched posteriorly; 4Rs shorter than 4M, slightly less than 0.75× 4M; third submarginal cell longer than either first or second submarginal cells, only slightly longer than second submarginal cell; 2m-cu not oblique, weakly curved along length, proximal 2rs-m by 4× vein width, nearly twice as long as 2Cu; 3Cu in medial position along 2Cu/2cu-a. Hind wing with short distal abscissa of Rs, longer than rs-m; without distal abscissa of M; 2M+Cu elongate, 1.75× 1M; jugal lobe absent.

Metasoma robust, maximum length as preserved 11 mm (paratype PE 1995/5243: 12 mm, PE 1995/5321: 10 mm); sting long, longer than metasomal sternum VI [visible in holotype and paratypes 5321 and 5234a: Figs 6a, c, d), valvulae of equal widths, thickened proximally at attachment to rami, rami thin, simple.

♂: *Latet*.

Timebombus Engel, subgen. nov.

Type species: *Bombus* (*Timebombus*) *palaeocrater* Engel et Wappler, sp. nov.

Diagnosis: Quite unlike *Calyptapis* and *Kronobombus*, the species of this subgenus has a narrow marginal cell, like that of extant lineages of *Bombus*. A further derived feature is the slightly oblique 2rs-m, with the anterior border of the third submarginal cell closer in length to the anterior border, and with 2rs-m straight anteriorly and weakly arched posteriorly without a prominent extension apically. It also has the bombine bowed 2Rs indicative of all *Bombus s.l.* Unlike more derived bombines, however, 2m-cu is long and weakly arched, rather than oblique and posteriorly extending more proximally as is the case in most extant *Bombus* and in the subgenus *Paraelectrobombus*. Unlike *Paraelectrobombus* the second medial cell is 'peaked' rather than 3M and 4M rather continuous, without an angle at their juncture. In extant subgenera of *Bombus* the second medial cell is about as broad apically (2m-cu) as it is proximally where it borders 2Cu, while in *Timebombus*, *Paraelectrobombus*, *Kronobombus*, and *Calyptapis* the second medial cell is distinctly broader apically. The hind wing is partly visible in the part of the holotype, with Sc+R particularly clear near the wing base and becoming fainter to trace as it extends apically; Rs, rs-m, 1M, M+Cu, cu-a, and A are faint but can be traced by following the margins of the tubular veins, although 1M+Cu and 1A are not complete as extending toward the base. Although incomplete and faint, it is noteworthy that the distal abscissa of M is absent and 2M+Cu is only slightly longer than 1M (Fig. 6k), the former consistent with all Bombini and the latter a feature similar to extant subgenera of *Bombus* (the membrane of the hind wing is not discernible). The malar space is apparently short, broader than long; the labrum with a faint medial furrow, broader basally and thin apically; and flagellomere I is as long as the combined lengths of flagellomeres II and III (Fig. 6i) (much like in *Mendacibombus*).

Etymology: The new genus-group name is a euphonious combination of the Middle English *time* (identical to the Modern English word), and the generic name *Bombus*. The gender of the name is masculine.

ZooBank LSID: urn:lsid:zoobank.org:act:F28E4B28-1CFB-4BE0-853B-46491F684BDE

Bombus (*Timebombus*) *palaeocrater* Engel et Wappler, sp. nov. (Figs 5, 6g-l; Supporting Information, Fig. S5c,d, S12)
Holotype (*hic designatus*): NHMMZ PE PE1997/6137a-LS and NHMMZ PE 1997/6137b-LS (Fig. 6h, i) (part and counterpart).
Type locality: Enspel, Rhineland-Palatinate, Germany.
Type stratum: Enspel Fm, Layer S16; digging site G10.
Age: 24.56±0.04 Ma, Chattian, Oligocene.
Etymology: The specific epithet is a combination of the Ancient Greek adjective *πᾰλαιός* (*palaiós*, meaning, "ancient") and the noun ***κρᾱτήρ*** (*krā̆tḗr*, meaning, "crater", "mouth of a volcano"), and refers to the crater lake in which the fossil was deposited.
ZooBank LSID: urn:lsid:zoobank.org:act:6F9C987C-ECD6-4498-AC28-8127A96F9177
Diagnosis: As for the subgenus (*vide supra*).

Description: ♀: Total body length (as preserved) 15 mm, forewing length (as preserved) ca. 7.7 mm. Integument sculpture and coloration not preserved [integument is represented by carbonaceous compression taphonomically altered; the bee's integument was likely black in life (like most bumble bees) but the dark coloration of preserved setae may or may not reflect an overall dark pubescence throughout the body]; body robust with abundant setae (where evident) [likely abundantly pubescent in life as in modern bumble bees]. Head apparently about as long as wide (as preserved), distinctly narrower than mesosoma [note that the head is slightly oblique as preserved, with the vertex tilted slightly backward, such that length of head is slightly foreshortened as seen, but degree to which the head may have been longer than wide is uncertain but if longer, then it would have been scarcely so; perhaps of proportions similar to species like *Bombus* (*Cullumanobombus*) *rufocinctus* Cresson or at most like medium-length bumble bees]. Antennal toruli apparently at head midlength, separated by a distance equivalent to torulus diameter; scape long, surpassing vertex; flagellomere I long, about as long as combined lengths of flagellomeres II and III (similar in this respect to *Mendacibombus*); clypeal furrow distinct, broad, nearly 0.2× labral length; labrum transverse, with faint medial furrow (likely shallow and weak in life), wide along proximal margin but quickly narrowing and thin apically; malar space apparently shorter than wide; mandible with apical margin curved (not oblique), with outer mandibular grooves present.

Mesosoma quite robust, maximum width as preserved 6.7 mm [propleura easily discernible in part]. Metatibia about as long as metatrochanter + metafemur; metatibia with corbicula [given that individual was obviously foraging the bee was likely a worker]; mesosoma nearly as long as metasoma (as preserved).

Forewing (Figs 6j, S5c), with membrane apparently hyaline, clear (Fig. 6g); 1M slightly proximal to 1cu-a, straight; 1Rs short, shorter than Rs+M; Rs+M longer than 2M; 2Rs bowed anteriorly; pterostigma slightly longer than wide, prestigma about as long as pterostigmal width; marginal cell narrow, apically slightly offset from anterior wing margin, apex narrowly rounded, with minute appendiculate stub, free portion of marginal cell shorter than portion bordering submarginal cells; r-rs about as long as 3Rs; 4Rs much longer than 3Rs; 1m-cu entering second submarginal cell in proximal third, thus 2M distinctly shorter than 3M; 3M angled at juncture with 4M (*i.e.*, second medial cell 'peaked'), shorter than 4M; 1rs-m straight; 2rs-m slightly oblique, straight anteriorly, weakly arched posteriorly, thus third submarginal cell only slightly extended apically in posterior section, 4Rs shorter than 4M, slightly more than 0.76× 4M; third submarginal cell longer than either first or second submarginal cells; 2m-cu not oblique, weakly curved along length, proximal 2rs-m by 2× vein width, much longer than 2Cu; 3Cu in medial position along 2Cu/2cu-a. Hind wing (Fig. 6k) with short distal abscissa of Rs, about as long as rs-m; 2M+Cu only slightly longer than 1M [hind wing largely missing except Sc+R extending from base to divergence of Rs, Rs extending to rs-m, with short distal abscissa Rs and no distal abscissa M, small portion of 1M present faintly and extending proximal for distance about equivalent to rs-m, then effaced in fossil].

♂: *Latet*.

Remarks: The bee is preserved lengthwise on its back, with the legs either tucked in under the body (forelegs and midlegs) or tightly alongside the body (hind legs). The bee's left wings are folded back over the body and therefore difficult to discern in the part and entirely obscured in the counterpart, the right forewing and hind wing are extended slightly obliquely away from the body making them more visible, at least in the part. The head is slightly tilted thereby appearing slightly foreshortened but nicely exposing the mandibles, labrum, and from an oblique slant the clypeus, exposing its apical furrow above the labrum. The outline of head and foreleg structures are nicely preserved in the counterpart, while there is more integument preserved in the part but given how black and taphonomically altered in the part many details of the head and mesosoma are less-easily observed. The part appears to show the ental surfaces of structures seen in ventral view in the counterpart.

Key to subgenera of *Bombus*

1. Forewing marginal tangent (*i.e.*, tangent from medioapical point on pterostigma to medioapical point of marginal cell apex) not crossed by length of 1rs-m within posterior of marginal cell: *e.g.*, Fig. S5b) [hind wing 2M+Cu, where known, elongate, 1.75× or more length 1M] 2

—. Forewing marginal tangent crossed by length of 1rs-m within posterior of marginal cell: *e.g.*, Fig. S5d) [hind wing 2M+Cu, where known, short, 1.5× or less length 1M] 3

2. Forewing prestigma shorter than pterostigmal width; 2rs-m sinuate owing to anterior curvature that reverses its sign posteriorly [Priabonian-Rupelian boundary] .... *Calyptapis* Cockerell

—. Forewing prestigma as long as pterostigmal width; 2rs-m straight anteriorly relative to posterior curvature [Chattian] .... *Kronobombus* Engel subgen. nov.

3. Forewing 2m-cu with posterior oblique relative to anterior; second medial cell with anterior border not 'peaked' or scarcely so 4

—. Forewing 2m-cu weakly curved across length; second medial cell with anterior border distinctly 'peaked' [Chattian] .... *Timebombus* Engel subgen. nov.

4. Forewing second medial cell distinctly broader apically than proximally (*i.e.*, 2m-cu significantly longer than 2Cu and also than third submarginal cell) [Aquitanian] .... *Paraelectrobombus* Nel & Petrulevičius

—. Forewing second medial cell about as broad apically as proximally (*i.e.*, 2m-cu about as long as 2Cu and frequently as broad as third submarginal cell apically) [Aquitanian to Recent] .... extant subgenera (refer to Williams *et al.*, 2008)

The transfer of *Oligobombus* to Electrobombini necessitates the following couplet for distinguishing these genera:

1. Forewing 4Rs much longer than 3Rs; r-rs longer than 3Rs; 3M less than 0.5× 4M [Bartonian] .... *Electrobombus* Engel

—. Forewing 4Rs subequal to 3Rs; r-rs about as long as 3Rs; 3M more than 0.5× 4M [Priabonian] .... *Oligobombus* Antropov

References

Auer W, Geier C, Bouchal JM, Grímsson F. **2024**. *Tilia americana*. *PalDat - A palynological database*.

Barden P, Engel MS. **2021**. Fossil social insects. In: Starr CK, ed. Encyclopedia of Social Insects. Cham, Switzerland: Springer, 384–403.

Beug HJ. **2004**. *Leitfaden der Pollenbestimmung für Mitteleuropa und angrenzende Gebiete*. München: Dr. Friedrich Pfeil.

Chandler MEJ. **1957**. The Oligocene flora of the Bovey Tracey lake basin, Devonshire. *Bulletin of the British Museum (Natural History) Geology* **3**: 72–123.

Dehon M, Engel MS, Gérard M, Aytekin AM, Ghisbain G, Williams PH, Rasmont P, Michez D. **2019**. Morphometric analysis of fossil bumble bees (Hymenoptera, Apidae, Bombini) reveals their taxonomic affinities. *ZooKeys* **891**: 71–118.

Dehon M, Michez D, Nel A, Engel MS, De Meulemeester T. **2014**. Wing shape of four new bee fossils (Hymenoptera: Anthophila) provides insights to bee evolution. *PLoS One* **9**: 1–16.

Duarte L. **1974**. Sobre uma flor de Bombacaceae da Bacia Terciária de Fonseca MG. *Anais da Academia Brasileira de Ciências* **46**: 407–411.

eFloras. **2008**.

Engel MS. **2001a**. A monograph of the Baltic amber bees and evolution of the Apoidea (Hymenoptera). *Bulletin of the American Museum of natural History* **2001**: 1–192.

Engel MS. **2001b**. Monophyly and extensive extinction of advanced eusocial bees: Insights from an unexpected Eocene diversity. *Proceedings of the National Academy of Sciences* **98**: 1661–1664.

Engel MS, Rasmussen C. **2021**. Corbiculate bees. In: Encyclopedia of Social Insects. Cham, Switzerland: Springer, 302–310.

Felder M, Weidenfeller M, Wuttke M. **1998**. Lithologische Beschreibung einer Forschungsbohrung im Zentrum des oberoligozänen, vulkano-lakustrinen Beckens von Enspel/Westerwald (Rheinland-Pfalz; Bundesrepublik Deutschland). *Mainzer geowissenschaftliche Mitteilungen* **27**: 101–136.

Ferguson DK. **1993**. Plant taphonomic studies with special reference to Messel. *Kaupia* **2**: 117–126.

Geier C, Ulrich S, Bouchal JM, Zetter R, Ngô Muller V, Jacobs BF, Uhl D, Grímsson F. 2025. Morphology and ultrastructure of Tilioideae pollen: how to differentiate *Craigia*, *Mortoniodendron*, and *Tilia*. *Botanical Studies* 66: 15. https://doi.org/10.1186/s40529-025-00463-1

Hall JW, Swain AM. **1971**. Pedunculate bracts of *Tilia* from the Tertiary of western United States. *Bulletin of the Torrey Botanical Club*: 95–100.

Hanes MM. **2015**. Malvaceae Jussieu. In: Committee F of NAE, ed. Flora of North America north of Mexico. New York, USA: Oxford University Press, 187–375.

Hazra T, Bera S, Khan MA. **2023**. First Fossil Mallow Flower from Asia. *International Journal of Plant Sciences* **184**: 106–121.

Herrmann M, Ashraf AR, Uhl D, Mosbrugger V. **2009**. Die oberoligozäne Palynoflora der Bohrung Enspel 1996/1 (Westerwald, W-Deutschland)–Teil 1: Sporen und Gymnospermen. *Mainzer geowissenschaftliche Mitteilungen* **37**: 33–76.

Herrmann M, Ashraf AR, Uhl D, Mosbrugger V. **2010**. Die oberoligozäne Palynoflora der Bohrung Enspel 1996/1 (Westerwald, W-Deutschland)–Teil 2: Angiospermen und Paläökologie. *Mainzer geowissenschaftliche Mitteilungen* **38**: 9–60.

(IBSCIB-CAS) I of B and SCI of B at the CA of S. **1982**. *Angiosperm pollen flora of tropical and subtropical China*. Science Press.

Köhler J, Uhl D. **2014**. *Die Blatt-und Karpoflora der oberoligozänen Fossillagerstätte Enspel:(Westerwald, Rheinland-Pfalz, W-Deutschland)*. Naturhistorisches Museum Mainz, Landessammlung für Naturkunde Rheinland-Pfalz.

Kvacek Z. **2004**. Early Miocene records of *Craigia* (Malvaceae sl) in the Most Basin, North Bohemia-whole plant approach. *Journal of Geosciences* **49**: 161–171.

Kvaček Z, Manchester SR, Zetter R, Pingen M. **2002**. Fruits and seeds of *Craigia bronnii* (Malvaceae - Tilioideae) and associated flower buds from the late Miocene Inden Formation, Lower Rhine Basin, Germany. *Review of Palaeobotany and Palynology* **119**: 311–324.

Mai DH. **1961**. Über eine fossile Tiliaceen-Blüte und tilioiden Pollen aus dem deutschen Tertiär. *Geol. Beih* **32**: 45–83.

Mai DH. **2000**. Die untermiozänen Floren aus der Spremberger Folge und dem 2. Flözhorizont der Lausitz. Teil III. Dialypetalae und Sympetalae. *Palaeontographica Abteilung B* **253**: 1–106.

Mai DH, Walther H. **1991**. Die oligozänen und untermiozänen Floren Nordwest-Sachsens und des Bitterfelder Raumes. *Abhandlungen des Staatlichen Museums für Mineralogie und Geologie zu Dresden* **38**: 1–230.

Manchester SR. **1992**. Flowers, fruits, and pollen of *Florissantia*, an extinct malvalean genus from the Eocene and Oligocene of western North America. *American Journal of Botany* **79**: 996–1008.

Manchester SR. **1994**. Inflorescence bracts of fossil and extant *Tilia* in North America, Europe, and Asia: patterns of morphologic divergence and biogeographic history. *American Journal of Botany* **81**: 1176–1185.

Mello CL, Sant'Anna LG, Bergqvist LP. **2000**. Sítio Paleontológico de Fonseca, Minas Gerais (Vegetais Fósseis do Terciário Brasileiro). In: Schobbenhaus C, Campos DA, Queiroz ET, Winge M, Berbert-Born M, eds. Sítios Geológicos e Paleontológicos do Brasil, 86. 73–79.

Michener CD. **2007**. *The bees of the world*. Baltimore: Johns Hopkins University Press.

Michez D, Vanderplanck M, Engel MS. **2012**. Fossil bees and their plant associates. In: Patiny S, ed. Evolution of plant-pollinator relationships. Cambridge, UK: Cambridge University Press, 103–164.

Olson, D. M., Dinerstein, E., Wikramanayake, E. D., Burgess, N. D., Powell, G. V., Underwood, E. C., ... & Kassem, K. R. (2001). Terrestrial Ecoregions of the World: A New Map of Life on Earth: A new global map of terrestrial ecoregions provides an innovative tool for conserving biodiversity. *BioScience*, *51*(11), 933-938.

Perveen A, Grafström E, El-Ghazaly G. **2004**. World Pollen and Spore Flora 23. Malvaceae adams. P.p. Subfamilies: Grewioideae, Tilioideae, Brownlowioideae. *Grana* **43**: 129–155.

Pigott D. **2012**. *Lime-trees and Basswoods - A biological monograph of the genus Tilia*. Cambridge, UK: Cambridge University Press.

Pingen M, Gregor HJ. **1994**. Neue Pflanzenfossilien aus den niederrheinischen Tertiär. VIII. *Tilia gieskei*. *Documenta naturae* **89**: 1–8.

Pingen M, Kvaček Z, Manchester RC. **2001**. Früchte und Samen von *Craigia bronnii* aus dem Obermiozän von Hambach (Niederrheinische Bucht: Deutschland). *Documenta naturae* **138**: 1–7.

Prokop J, Dehon M, Michez D, Engel MS. **2017**. An early Miocene bumble bee from Northern Bohemia (Hymenoptera, Apidae). *ZooKeys* **710**: 43–63.

POWO. **2024**. Plants of the World Online. Facilitated by the Royal Botanic Gardens, Kew.

De Saporta G. **1862**. Études sur la végétation du sud-est de la France à l'époque tertiaire. *Annales des sciences naturelles. Botanique* **17**: 191–311.

De Saporta G. **1877**. Le Périodes Végétales de L'Epoque Tertiaire. In: Journal Hebdomadaire Illustre. Paris, France: Masson, 403–408.

Schultz TR, Engel MS, Aschier JS. **2001**. Evidence for the origin of eusociality in the corbiculate bees (Hymenoptera: Apidae). *Journal of the Kansas Entomological Society* **74**: 10–16.

Spicer RA. **1989**. The formation and interpretation of plant fossil assemblages. In: Advances in botanical research. Elsevier, 95–191.

Spicer RA. **1991**. Plant taphonomic processes. In: Allison PA, Briggs DEG, eds. Topics in Geobiology. Volume 9. Taphonomy: Releasing the data locked in the fossil record. New York: Plenum Press New York, 71–113.

Uhl D. **2015**. Preliminary note on fossil flowers and inflorescences from the late Oligocene of Enspel (Westerwald, W-Germany). *Palaeobiodiversity and Palaeoenvironments* **95**: 47–53.

Ya T, Gilbert MG, Dorr LJ. **2007**. Tiliaceae. In: Wu ZY, Raven PH, Hong DY, eds. Flora of China. Beijing. St Luis, MO: Science Press. Missouri Botanical Garden Press., 240–263.

Wappler T, De Meulemeester T, Murat Aytekin A, Michez D, Engel MS. **2012**. Geometric morphometric analysis of a new Miocene bumble bee from the Randeck Maar of southwestern Germany (Hymenoptera: Apidae). *Systematic Entomology* **37**: 784–792.

Williams PH, Cameron SA, Hines HM, Cederberg B, Rasmont P. **2008.** A simplified subgeneric classification of the bumblebees (genus *Bombus*). *Apidologie*, *39*(1), 46-74.

Zetter R, Weber M, Hesse M, Pingen M. **2002**. Pollen, pollenkitt, and orbicules in *Craigia bronnii* flower buds (Tilioideae, Malvaceae) from the Miocene of Hambach, Germany. *International Journal of Plant Sciences* **163**: 1067–1071.
